# Modulation of Biomolecular Aggregate Morphology and Condensate Infectivity

**DOI:** 10.3390/biom16040492

**Published:** 2026-03-25

**Authors:** Josephine C. Ferreon, Kyoung-Jae Choi, My Diem Quan, Phoebe S. Tsoi, Cristopher C. Ferreon, Ulas Coskun, Shih-Chu Jeff Liao, Allan Chris M. Ferreon

**Affiliations:** 1 Department of Biochemistry and Molecular Pharmacology, Baylor College of Medicine, Houston, TX 77030, USAmy.quan@bcm.edu (M.D.Q.);; 2 ISS, Inc., 1602 Newton Drive, Champaign, IL 61822, USA; ucoskun@illinois.edu (U.C.);

**Keywords:** hnRNPA1, prion-like domain, LLPS, biomolecular condensates, aggregation, fibrillation, phase transitions, Alzheimer’s disease, ALS, FLIM

## Abstract

Neurodegenerative diseases feature diverse pathological protein aggregates, including Lewy bodies in Alzheimer’s disease (AD) and skein-like filaments in amyotrophic lateral sclerosis (ALS). The physical mechanisms underlying this morphological diversity remain unclear. Here, we demonstrate that aggregation of the prion-like domain of hnRNPA1 (A1PrD), implicated in AD and ALS, is driven by solution composition and phase transition dynamics. Utilizing 3D timelapse and fluorescence lifetime imaging microscopy, we show that solution conditions modulate phase separation, gelation, and fibrillation, resulting in distinct structures such as fibril, gel, and starburst morphologies. Homotypic and heterotypic interactions between A1PrD and RNA were observed to shift the balance between pathological and physiological condensates. Importantly, amyloid-rich starbursts displayed prion-like infection capabilities toward amyloid-poor condensates. Our findings highlight how the interplay between solution composition and kinetic balances of liquid-liquid phase separation, gelation, and fibrillation shapes the diverse pathological aggregate morphologies characteristic of neurodegenerative diseases.

## 1. Introduction

The formation of protein aggregates is a hallmark of various neurodegenerative diseases (NDs), characterized by morphological diversity ranging from amorphous aggregates to highly structured fibrillar assemblies. Protein aggregates found in diseased tissues exhibit distinct morphologies. Lewy bodies, prominent in Alzheimer’s disease (AD), typically manifest as circular structures featuring a dense protein core measuring up to 20–30 µm in diameter [1], surrounded by radiating filaments [2,3]. Amyotrophic lateral sclerosis (ALS), characterized by progressive neuron degeneration [4,5], presents unique histopathological features such as skein-like inclusions (SLIs) and hyaline inclusions (HI). HI resemble Lewy bodies, appearing as round, glassy structures with central cores ranging from 2 to 20 µm in diameter, often with peripheral halos, while SLIs are composed of thick filamentous bundles extending up to 40 µm in length [6]. Recently, star-shaped inclusions have been reported for TDP-43 in LATE diseases [7], highlighting additional morphological complexity. Despite extensive research, the precise mechanisms underlying these morphological variations remain unclear. Since the seminal study with FUS, it has been proposed that protein aggregation can also occur through liquid-liquid phase separation (LLPS) [8,9,10], wherein a condensed liquid phase separates from the dilute solution [11,12,13,14,15]. Aging and maturation of these condensates can trigger a liquid-to-solid phase transition. Subsequently, several proteins linked to neurodegeneration, including Tau, HTT, α-synuclein, TDP-43, and hnRNPA1, have been shown to form fibrils under LLPS-promoting conditions [12,16,17,18,19]. By significantly increasing local protein concentration, LLPS can accelerate the aggregation process [13,19,20,21]. However, few studies have addressed the relationship between LLPS-mediated aggregation and classical aggregation pathways studied under non-LLPS conditions. Here, we explored hnRNPA1, a ribonucleoprotein implicated in pathological inclusions in AD and ALS, known for its propensity toward LLPS and fibrillar aggregation [12,22,23,24,25,26]. We examined how solution conditions affect the formation of hnRNPA1 aggregates, particularly FUS-like starbursts or thin fibrillar morphologies. Using timelapse confocal 3D microscopy and fluorescence lifetime imaging microscopy (FLIM), we tracked the morphological evolution of hnRNPA1 prion-like domain (A1PrD) condensates. Our observations revealed that liquid gel-like starbursts can ‘infect’ younger condensates through direct coalescence with phase-matched droplets, exacerbating the formation of starburst morphologies. Our findings demonstrate that solution state and composition substantially influence condensate morphology, dynamics, and amyloid formation, emphasizing the critical interplay between condensate fluidity, gelation, and fibrillation in generating diverse aggregate morphologies.

## 2. Materials and Methods

### 2.1. Cloning

The C-terminal hnRNPA1 sequence (residues 186-320; A1PrD) was PCR-amplified from pET9d-hnRNPA1 (Addgene plasmid #23026, gift from Douglas Black, UCLA, Los Angeles, CA, USA) to generate A1PrD (Prion-like Domain of hnRNPA1) plasmid constructs. The primers used in this study are as follows: 5′-ctgtattttcagggacatatggctagtgcttcatccagcc-3′ and 5′-ctcgagtgcggccgcaccttaaaatcttctgccactgcc-3′. Purified PCR product and NdeI/HindIII-digested pET29-mCherry-TEV vector (developed in-house) were used for Gibson assembly [27]. The reactions consisted of 4 µL 2X Gibson Mixture (New England Biolabs, Ipswich, MA, USA) and 4 µL insert-linearized vector mixtures (7-fold molar excess of insert), incubated at 50 °C for 1 h. The complete mixtures were used to transform chemically competent DH5α cells. The plasmid (pET28-mCherry-A1PrD) containing the insert was verified by DNA sequencing.

### 2.2. Protein Expression and Purification

The pET28-mCherry-A1PrD plasmid was transformed into *E. coli* Rosetta (DE3) competent cells (Novagen, Houston, TX, USA) and grown at 37.0 °C in Terrific Broth medium containing kanamycin. Cells were induced for expression with 1 mM IPTG at OD600 1.0-1.5. Growth was allowed to continue overnight at 18.0 °C, and cells were subsequently harvested by centrifugation. The recombinant fusion protein, N-terminal 6XHis-mCherry-TEV cleavage site-A1PrD, was expressed as a soluble protein (MW 45.5 kDa). For A1PrD purification, cells were suspended in purification buffer (300 mM NaCl, 50 mM Tris, pH 7.5) containing a protease inhibitor cocktail (GenDEPOT, Katy, TX, USA), and lysed using a homogenizer (Avestin, Ottawa, ON, CAN). Purifications were then carried out either by using a batch/gravity method wherein clarified lysates were applied to HisPur cobalt resin (ThermoFisher Scientific, Waltham, MA, USA) or by FPLC using Talon columns (GE Healthcare, Chicago, IL, USA). After extensive washing, 6XHis-tagged protein was eluted using 200 mM imidazole, dialyzed against purification buffer, and cleaved with TEV protease (prepared in-house; ~1:50 mass ratio) to remove 6XHis-mCherry tags. The cleaved A1PrD protein was then separated from the 6XHis-mCherry tags by passing the sample through the cobalt resin again. A1PrD fractions were further purified by reverse-phase HPLC (20–60% ACN gradient with 0.1%TFA) using semi-preparative C3 or C18 columns (Agilent, Santa Clara, CA, USA).

### 2.3. Protein Fluorescence Labeling

Purified A1PrD were dye-labeled using NHS ester chemistries with Alexa Fluor 488 (A488) or Alexa Fluor 647 (A647) NHS (with 1:10 protein/dye labeling ratio, incubated at RT for 1–3 h; Thermo Fisher Scientific), similar to previously described methods [28,29]. The labeled samples were subsequently purified by reverse-phase HPLC (20–60% ACN gradient with 0.1%TFA), and lyophilized and stored at -80 °C until further use.

### 2.4. Fluorescence Microscopy Imaging

Confocal fluorescence imaging was performed using LSM710 or LSM880 confocal microscopes (Zeiss, Oberkochen, Germany) for timelapse z-stack experiments. DIC and epifluorescence imaging were performed using a Nikon Ti2 SE microscope (Nikon, Tokyo, Japan) and EVOS FL Imaging System (Thermo Fisher Scientific). To obtain microscopy phase diagrams for varying [NaCl] and RNA concentrations, fluorescence imaging was performed using a spinning disk Nikon Ti2 SE microscope (Nikon) equipped with a CSU-W1 confocal scanner unit (Yokogawa, Tokyo, Japan).

### 2.5. Sample Preparation for Fluorescence Imaging

For PEG- or RNA-mediated LLPS experiments, separate stock solutions of unlabeled (880 µM) and labeled A1PrD (29 µM; either A647- or A488-labeled) were prepared and dissolved in 4 M urea, 200 mM NaCl, αβγ buffer (10 mM sodium acetate, 10 mM sodium phosphate, 10 mM glycine, pH 7.5). Substock solutions of protein mix (1:300 labeled vs. unlabeled) were prepared and frozen in 1–2 µL aliquots at -80 C prior to imaging. Each aliquot was diluted to 20–40 µL volume with other experimental buffer components, generating the final solution conditions for A1PrD (~1:300 labeled to unlabeled ratio; 67 nM labeled:20 µM unlabeled A1PrD) in 100 mM urea, 200 mM NaCl, αβγ buffer, either with 10 µM non-specific RNA (5′-GGGCCCCCGGGUACCGAGCUGCUAAUCAAAACAAAACAAAAGCU-3′; described in Molliex et al. [12]) or 10% *w/v* PEG-8K (Hampton Research, Aliso Viejo, CA, USA). For some samples, 3 µM thioflavin T (ThT) was added to monitor amyloid formation.

For experiments in different NaCl and RNA conditions, lyophilized A1PrD protein was dissolved in water and subsequently mixed with stock αβγ buffer containing various NaCl and RNA concentrations to generate the final solution conditions ([NaCl]: 0, 200, and 400 mM; and [RNA]: 0, 1, and 10 µM); pH was individually adjusted for each stock buffer. In some cases, either A647- or A488-labeled A1PrD were added at a 1:100 or 1:200 labeled to unlabeled ratio (~100 or 200 nM labeled:20 µM unlabeled A1PrD), or A488-RNA (~50 nM) was added to 10 µM unlabeled RNA (1:200 labeled to unlabeled ratio). The different protein concentrations used in experiments are indicated in the figure legends.

Unless specified otherwise, samples were deposited onto a glass-bottomed microscope dish (MatTek dish, or Ibidi µ-Dish, 35 mm, with or without grid). To minimize evaporation effects in experiments performed at RT, several 20 µL buffer droplets were added around the dish periphery, and dishes were sealed with parafilm. For thermal experiments, samples were covered in paraffin oil or a cover slip (with a diameter bigger than the dish bottom coverslip leaving enough height (~1 mm) for molecular movement within the sample), which was then sealed with nail polish.

Four-dimensional (timelapse z-stack) confocal imaging. Timelapse z-stack images were collected spanning 60–100 µm above the glass surface and from 10 min to 9 h of collection at varying time intervals (0 to 10 min per z-stack). For the timelapse z-stack experiment at 55 °C, 100 µL samples of A647- or A488-labeled A1PrD (1:300 labeled to unlabeled ratio; 67 nM labeled:20 µM unlabeled A1PrD) with 10 µM RNA in 200 mM NaCl and αβγ buffer were pipetted onto 35 mm (No. 1.5 coverslip) MatTek dishes (MatTeK Corporation, Ashland, MA, USA) covered with CoverwellTM chambers (Grace Bio-labs, Bend, OR, USA) and sealed with parafilm to prevent evaporation.

LLPS and starburst reversibility experiments using temperature. A647-labeled A1PrD (1:300 labeled to unlabeled ratio; 67 nM A647-labeled:20 µM unlabeled A1PrD) with 10 µM RNA in 200 mM NaCl and αβγ buffer was pipetted onto a MatTek glass-bottom dish (MatTeK Corporation) pre-coated with Sigmacote (Sigma-Aldrich, St. Louis, MO, USA). Paraffin oil was added to prevent evaporation of the sample. The dish was incubated for ~2 min at temperatures from 25 to 60 °C with 5 °C intervals under thermal control using a Tokai Hit ThermoPlate (ThermoFisher Scientific). The heating rate was about 6–12 s/°C. The sample was then cooled back to 25 °C. Different aged samples were prepared (1 h and 4 h, RT) and subjected to the same heating and cooling process described above to verify the reversibility of the condensed droplets or the aged starbursts.

For the timelapse video of A1PrD temperature reversibility (Movie S11), A488-labeled A1PrD (1:300 labeled to unlabeled ratio; ~70 nM A488-labeled:20 µM unlabeled A1PrD) with 10 µM RNA in 200 mM NaCl and αβγ buffer was applied to a CELLview glass-bottom slide (Greiner bio-one) pre-coated with Sigmacote (Sigma-Aldrich). Paraffin oil was added to prevent evaporation of the sample. The dish was incubated at 25 °C for 3 min under thermal control using a Tokai Hit ThermoPlate (Tokai Hit Co., Shizuokaken, Japan). The sample was heated up to 60 °C, which took 90 s. After an additional 210 s of incubation, the sample was cooled down to 25 °C in 90 s. The temperature for each recorded image was noted.

LLPS and starburst reversibility experiments using 1,6-hexanediol. A647-labeled A1PrD (1:300 labeled to unlabeled ratio; 67 nM A647-labeled:20 µM unlabeled A1PrD) was combined with 3 µM ThT and 10 µM RNA in 200 mM NaCl and αβγ buffer in a Grid-50 glass-bottom µ-Dish (Ibidi) pre-coated with Sigmacote. Four aged samples (1, 3, 6 and 23 h) were prepared at RT. After incubation, hexanediol was added to a final concentration of 10% (*v*/*v*), followed by another 1 h of incubation. Samples were imaged before and after hexanediol treatment.

Gel and starburst reversibility experiments using 1% (*w/v*) SDS. A488-labeled A1PrD (~1:300 labeled to unlabeled ratio; ~70 nM A647-labeled:20 µM unlabeled A1PrD) was prepared in αβγ buffer with various [NaCl] and [RNA] concentrations in a 384-well glass-bottom plate (Greiner sensoplate plus). The samples were aged for 48 h. Timelapse videos were immediately taken 1 min after the addition of SDS (to 1% (*w/v*) final concentration) with 30 s intervals for 5 min. After 90 min, the SDS solution was replaced with buffer solution containing Amytracker 480 (1:200 dilution of company stock solution; Ebba Biotech). All images were collected using the spinning disk confocal microscope (Nikon).

### 2.6. LLPS Phase Diagrams

LLPS was quantified by UV light scattering measurements at 350 nm using Nanodrop 2000 (Thermo Fisher Scientific). Lyophilized A1PrD (20 and 40 µM final concentrations) dissolved in water was mixed with buffer containing various salt and RNA concentrations to generate the final solution conditions ([NaCl]: 0, 200, and 400 mM; and [RNA]: 0, 1, and 10 µM). UV measurements were recorded after 2 min of sample incubation.

### 2.7. Partition Efficiency Experiments

Partition efficiency of RNA and A1PrD inside and outside condensates in selected [NaCl]-[RNA] (mM-mM) conditions (i.e., 0-0, 0-10, 400-0, and 400-10) was determined from protein and RNA band intensities quantified from SDS-PAGE and Native PAGE (without SDS in running buffer) gels, respectively. Samples were incubated for 30 min and centrifuged for 30 min. Concentrations inside condensates were calculated from the band intensities of the pellet fractions. Concentrations outside condensates (supernatant fraction) were calculated by subtracting the pellet from the total sample concentrations (included as reference standards). Partition efficiency was calculated as the ratio of inside vs. outside condensate concentrations.

Quantification of soluble and insoluble (1,6-hexanediol- and SDS-treated) fractions. 20 µM A1PrD in various solution conditions ([NaCl]: 0, 200, and 400 mM; and [RNA]: 0, 1, and 10 µM) was prepared and incubated for 30 min and 48 h at RT. Quantification of soluble fractions: After an initial 30 min of incubation, the supernatant was obtained after 30 min centrifugation at 17,000× *g* and mixed with an equal volume of SDS loading dye (2% SDS). After heating at 95 °C for 5 min, samples were loaded onto 4–20% pre-cast Mini-PROTEAN Tris-Glycine gel (TG; Bio-Rad, Hercules, CA, USA) and electrophoresed for 25–45 min at 160 mV RT in 1x TGS buffer (vWR). Quantification of hexanediol- and SDS-insoluble fractions: After 48 h of incubation, samples were mixed with an equal volume of 20% hexanediol (in water) or 2% SDS (with 20% glycerol, 20 mM Tris, pH 8). The mixtures were incubated for 15 min at RT and centrifuged for 30 min at 17,000× *g*. The pellets were dissolved in 2X SDS loading buffer (2% SDS), heated at 95 °C for 5 min, and vortexed. Samples were run in SDS-PAGE gels as described above. Band intensities were quantified using ImageLab software ver. 5.1 build 8 (Bio-Rad).

### 2.8. Image Analysis

Analysis and processing of imaging data were performed using ImageJ ver. 1.52 (NIH, Washington, DC, USA), Zen ver. 2.3 blue edition (Zeiss), Imaris ver. 9.2 (Bitplane, South Windsor, CT, USA) and NIS elements ver. 5.42.06 build 1821 (Nikon) software. Droplets imaged at early timepoints (within 30 min) were analyzed for size and aspect ratios using the Analyze Particles Plugin in ImageJ. Other measurements such as filament lengths and thickness, droplet core diameters, intensity sum and surface volumes were analyzed using Imaris. Movies of raw data and surface-rendered images were generated using Imaris. Normalized ThT intensity (sum of fluorescence intensity divided by surface volume) of the aging droplets was fitted to an exponential model {y = y_0_ + Ae^(−x/t_1/2_)}, where y_0_ is the normalized ThT intensity at time 0 and A is the signal amplitude.

### 2.9. Fluorescence Recovery After Photobleaching (FRAP)

FRAP imaging was performed using a Zeiss LSM880 laser-scanning confocal microscope system with a 40× objective for selected sample conditions using 200 nM A647-labeled:20 μM unlabeled A1PrD and A488-labeled f1 RNA in different [NaCl]-[RNA] (mM-mM) conditions (i.e., 0-10, 400-0, and 400-10). Different regions of interest (ROI; ~2 μm diameter spots) were selected, and the reference ROIs were drawn in adjacent regions. Following 2–3 baseline images, ROIs were bleached for 200 iterations at 100% laser power (633 and 488 nm) and were imaged for up to 4–6 min post bleaching to calculate fluorescence recovery. FRAP recovery curves were corrected for background photobleaching (reference ROIs) and normalized against pre-bleach intensity values.

### 2.10. Transmission Electron Microscopy (TEM)

Negative-stain TEM images were collected using a Hitachi H7500 microscope with an 80 kV accelerating voltage, equipped with an AMT XR-16 digital camera at 4000–40,000× magnification. The 300-mesh copper grids (FCF300-Cu, Electron Microscopy Sciences) were incubated with protein or protein-RNA samples in various conditions (with 10% *w*/*v* PEG-8K or 10 µM f1 RNA) at varying timepoints (30 min to 19 h). For the temperature-induced aggregation experiments, the mesh grids were soaked with the protein samples in a PCR tube and incubated at 55 °C using a PCR machine (ProFlex, ThermoScientific) for 1 and 4 h, respectively. Afterwards, the grids were washed three times by soaking in distilled water for 30 s, and then negatively stained with 1% uranyl acetate for 0.5–3 min, followed by washing with distilled water three times. Additional TEM images were taken using JEOL 1400+ TEM and imaged at 80 kV using an AMT XR16 mid-mount camera. Aged samples (48 h) with different NaCl and RNA concentrations were dropped onto formvar and carbon-coated copper slot grids and allowed to sit for 2 min. The grids were wicked off with lint-free filter paper. Sample grids were negatively stained using 2% aqueous phototungstic acid via the drop method onto each grid and allowed to sit for 2 min. The grids were wicked off again with lint-free filter paper. Grids were allowed to dry overnight.

### 2.11. FLIM Instrumentation and Experiments

Fluorescence lifetime imaging microscopy (FLIM) experiments were conducted at pH 7.5 and RT (~21.5 ± 1 °C) using a custom-built ISS Alba confocal laser microscopy system that employed an Olympus IX81 microscope equipped with an Olympus 60×/1.2 NA water objective lens, galvo-controlled mirrors, and imaging and FastFLIM^TM^ modules (ISS). Instrument calibration was carried out using rhodamine 110 dye in water (4.0 ns lifetime). Samples were prepared as described above for general fluorescence imaging measurements. For the different NaCl and RNA combination experiments, a 1:200 labeled to unlabeled ratio (200 nM A488-labeled:20 µM unlabeled A1PrD) was used. Frequency domain FLIM data were analyzed using phasor analysis [30] and FD lifetime fitting (Figure S3). For FD fitting, FLIM images were fitted with a binning size of 3 × 3 pixels to 5 different modulation frequencies. An example of a FLIM FD fit and corresponding residual plot is shown in Figure S3. The laser power utilized is typically within 5–50 µW. Measured fluorescence lifetimes were found to be independent of the laser powers used (5–50 µW, Figure S4). We also checked that the lifetime values were independent of binning size and z-slices (Figure S5). Confocal FLIM images and phasor plots were generated using the PhasorAnalysis module in VistaVision software V4.2 (ISS, Inc., Champaign, IL, USA). FLIM histograms were generated using VistaVision V4.2 (ISS) and sometimes re-plotted using OriginPro 9.8.5.212 software (OriginLab, Northampton, MA, USA).

### 2.12. Generating Phasor Plots

Phasor analysis is a means of presenting fluorescence decay as pixels of an image. Using phasor representation, different molecular species can be observed through the clustering of pixels on specific regions of the phasor plot. Briefly, data at each pixel are transformed through Equations (1):

(1) $$\begin{array}{c} g_{i,j}\left(\omega \right)=m\cos\left(\varphi \right)\\ s_{i,j}\left(\omega \right)=m\cos\left(\varphi \right) \end{array}$$

where at each modulation frequency (ω), FLIM data consist of both the phase delay (φ) and amplitude modulation ratio (m) and the indexes i and j identify a pixel of the original image.

The values of this transformation are then plotted on a two-dimensional histogram or phasor plot, where each pixel of the FLIM image is represented as a point in the plot. For single-lifetime species, lifetime can be directly transformed by Equations (2):

(2) $$\begin{array}{c} g_{i,j}=1/(1+\omega^{2}\tau_{i,j}^{2})\\ s_{i,j}=1/(1+\omega^{2}\tau_{i,j}^{2}) \end{array}$$

where τ is the frequency decay of the species. The two coordinates of a phasor representing a single-lifetime species have a relationship:

(3) $${\left(g-0.5\right)}^{2}+\;s^{2}=0.25$$

From the phasor plot, the lifetime of a species can be determined by its coordinate value.

### 2.13. Quantification of Fluorescence Lifetime Clusters

FLIM images were fitted with a binning size of 3 × 3 pixels to 5 different modulation frequencies to obtain fluorescence lifetimes for the various conditions. For the dilute conditions (non-LLPS conditions), images were taken in the absence of unlabeled protein. For different [NaCl]-[RNA] (mM-mM) conditions (i.e., 0-0, 0-10, 400-0, and 400-10), fluorescence lifetimes were calculated from the region averaged values considering only the condensed phases (intensities >300 au). For RNA- and PEG-modulated LLPS conditions, fluorescence lifetimes were calculated from multiple images grouped and classified based on their incubation time and morphology. For PEG-induced LLPS: early droplets (10–30 min incubation), aged droplets (>30 min), and starbursts (>5–24 h, and presence of filaments). For RNA-enhanced LLPS: early droplets (10–30 min incubation), initiating or early starbursts (<3 h and presence of filaments), and late starbursts (>5–24 h and presence of filaments). To generate statistics from multiple heterogeneous images, fluorescence lifetimes for each group were clustered into four lifetime bins: <2.1, 2.1–2.45, 2.45–2.8 and >2.8 ns. The bins were based on observed distinct peaks for representative morphologies (Figures S9–S11 and S16–S18).

### 2.14. Disorder Prediction and pI Calculation

hnRNPA1 amino acid sequences correlating to NTD, RRM1, RRM2, and CTD were submitted to ProtParam [31] where theoretical isoelectric points were computed. Domain disorder scores were determined from IUPRED [31,32,33].

## 3. Results

**Figure 1:**
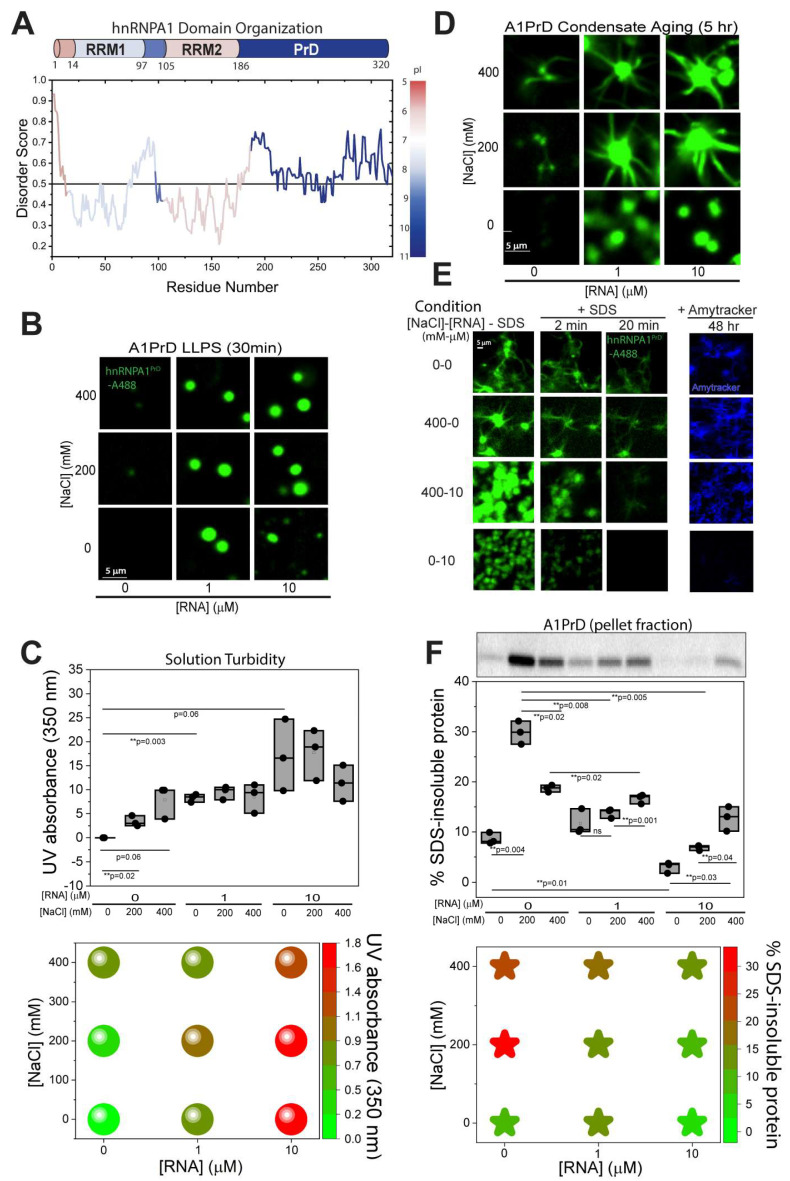
Homotypic and heterotypic interactions modulate A1PrD LLPS and aggregate morphology. (**A**) Domain structure of hnRNPA1 featuring two RNA recognition motifs (RRMs) and a C-terminal prion-like domain (PrD). Disorder prediction was performed using IUPRED2A [35], with scores above 0.5 indicating increased disorder. The color gradient reflects calculated isoelectric points (pI) of protein segments. (**B**) Confocal fluorescence images of hnRNPA1 PrD (A1PrD; 20 µM unlabeled protein with 100 nM A1PrD-A488) at various concentrations of NaCl and RNA in αβγ buffer (10 mM sodium acetate, 10 mM sodium phosphate, 10 mM glycine, pH 7.5) after a 30 min incubation period. Note that conditions with 0 mM NaCl and 10 µM RNA exhibited significant surface wetting not depicted in images. (**C**) LLPS of A1PrD monitored by UV light scattering at 350 nm (top panel, n = 3) at varying concentrations of NaCl and RNA in αβγ buffer. Data are also represented as a NaCl-RNA phase diagram (bottom panel). (**D**) Fluorescence images of samples from (**B**) following 5 h of incubation. (**E**) Selected conditions ([NaCl]-[RNA] in mM-µM: 0-0, 400-0, 400-10, and 0-10) after 48 h of aging. Fluorescence images were captured before (first column) and after adding 1% (*w/v*) SDS (2 and 20 min, second and third columns, respectively; see Movies S1 and S2 for timelapse videos of 0-10 and 400-0 conditions). The final column displays fluorescence images after incubation with Amytracker 480 (blue) in 1% SDS for 90 min. (**F**) Quantification of SDS-insoluble fractions under various [NaCl]-[RNA] conditions after 48 h, assessed via SDS-PAGE (top panel). The visualization plot illustrates fibril formation intensity (bottom panel; green indicating lowest and red highest intensity). Statistical significance determined by two-sided paired Student’s *t*-tests; with and ** *p* < 0.01.

Solution composition strongly influences the morphology of aggregates formed by hnRNPA1 and its prion-like domain (A1PrD). Unless noted otherwise, 20 µM A1PrD was used as a common working concentration because it robustly reported phase behavior across the NaCl-RNA matrix while enabling direct comparison of droplet, gel, and fibril morphologies across microscopy, FLIM, FRAP, and TEM experiments. Early timepoints (<2 h) were used to capture initial condensation before extensive aging, whereas later timepoints (>5–24 h) were used to evaluate matured gel-like and fibrillar states. Full-length hnRNPA1 contains two RNA recognition motifs (RRMs) and a highly basic, intrinsically disordered C-terminal PrD known to readily undergo aggregation and engage in non-specific, heterotypic interactions with RNA [12] (Figure 1A). Previously, we demonstrated that A1PrD phase separation is sensitive to physicochemical conditions, including pH, protein and NaCl concentrations, and the presence of RNA [26]. To further elucidate how LLPS correlates with aggregate formation, we systematically examined A1PrD condensation and amyloidogenesis under varying NaCl and RNA levels and protein concentrations (Figure 1B–F and Figure S1). LLPS was monitored via confocal fluorescence microscopy using Alexa Fluor 488-labeled A1PrD (A488-A1PrD, Figure 1B) and UV light scattering (Figure 1C). At the 30 min timepoint, we observed micron-sized (>2 µm) droplets in conditions containing RNA (Figure 1B). Increasing RNA from 1 to 10 µM enhanced LLPS, consistent with prior studies [25]. NaCl effects were more complex: in the absence of RNA, increasing NaCl (0 to 400 mM) promoted LLPS, likely due to electrostatic screening of repulsive homotypic interactions between A1PrD molecules. However, at high RNA concentrations (10 µM), elevated salt reduced LLPS, possibly by disrupting favorable heterotypic A1PrD-RNA interactions (Figure 1C). The condition with 0 mM NaCl and 10 µM RNA (condition 0–10) exhibited the most pronounced LLPS, characterized by droplet fusion and surface wetting. The absence of salt may have preserved strong electrostatic attraction between the positively charged A1PrD and negatively charged RNA. After 5 h, aggregate morphologies diverged significantly across conditions (Figure 1D). In most conditions that showed robust LLPS at 30 min, we observed the emergence of “starburst” morphologies (aggregates with a near-spherical dense core and radiating fibrillar extensions), like those previously described for FUS and hnRNPA1/B1 PrDs [11,23]. Interestingly, the 0-0 condition (no salt, no RNA) initially showed no detectable condensates at the micron scale, but at over 24–48 h, irregular filamentous aggregates emerged (Figure 1E), some displaying dense cores reminiscent of starbursts. Unexpectedly, no starbursts were detected in conditions 0-1 and 0-10 (0 mM NaCl with 1 or 10 µM RNA), despite their high LLPS propensity. This suggests that strong heterotypic A1PrD-RNA interactions may inhibit starburst formation. These findings are consistent with models proposing that RNA or other nucleic acids can modulate aggregation by buffering or redirecting the aggregation pathway [34], potentially preventing the structured growth associated with starburst morphologies.

To probe the properties of condensate cores and quantify amyloid fibril content, we applied 1% (*w*/*v*) SDS, a detergent known to selectively disrupt weak, reversible interactions while sparing more stable, solid-like fibrillar assemblies. Representative conditions from the [NaCl]-[RNA] matrix [0-0, 0-10, 400-0, 400-10] were evaluated. Within 2–20 min of SDS treatment, we observed rapid dissolution of the dense condensate cores or starburst centers, whereas most peripheral fibrils remained intact (Figure 1E; Movies S1 and S2). To independently confirm the presence of amyloid fibrils, we added Amytracker 480 dye at 90 min. Fibril-specific fluorescence revealed substantial amyloid formation in conditions 0-0, 400-0, and 400-10, but not in 0-10, indicating reduced fibrillogenesis under high-RNA/low-salt conditions. Quantitative analysis of SDS-insoluble fractions by SDS-PAGE further supported these findings, with lower levels of detergent-resistant material in the high-LLPS condition (0-10; Figure 1F and Figure S2). Taken together, these results reveal an anti-correlation between LLPS propensity and amyloid fibril formation (compare Figure 1C,F, bottom panels; Figure S2), suggesting that highly dynamic liquid-like condensates may buffer or delay the transition to irreversible fibrillar structures.

**Figure 2:**
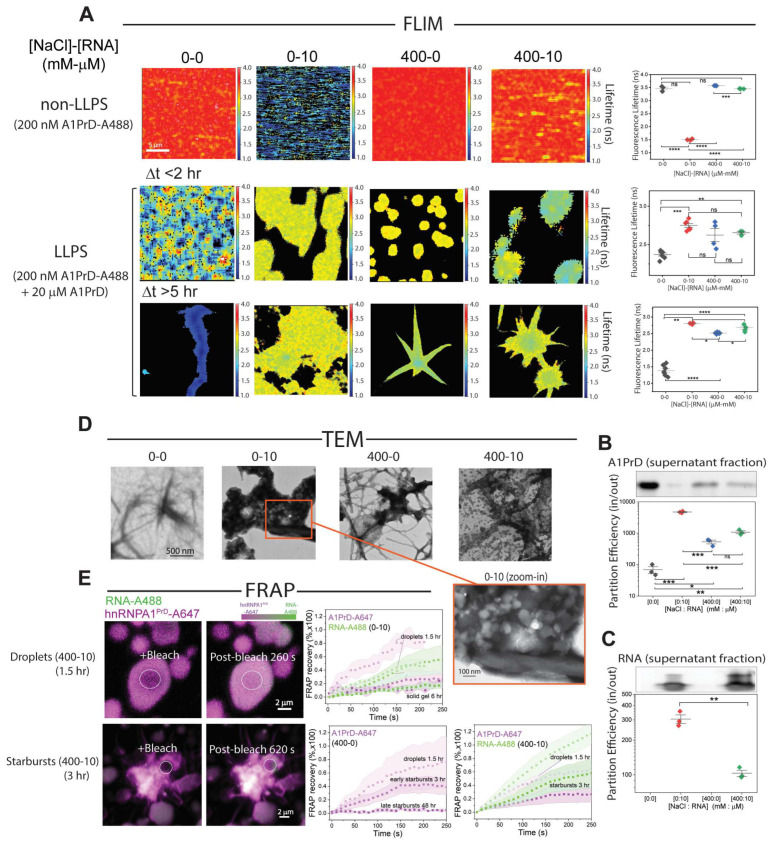
Distinct microenvironments within homotypic and heterotypic condensates. (**A**) Fluorescence lifetime imaging microscopy (FLIM) of A1PrD under selected NaCl-RNA conditions ([NaCl]-[RNA], mM-µM: 0-0, 0-10, 400-0, and 400-10), comparing non-LLPS controls (without unlabeled protein) with LLPS samples at early and late timepoints. Corresponding intensity images are provided in Figure S6. Right panels show quantified FLIM lifetimes (mean ± SD; n = 3–7 images per condition). (**B**) Partition efficiency of A1PrD in condensates (inside vs. outside) quantified by SDS-PAGE analysis of supernatant fractions across varying NaCl-RNA conditions (top panel; n = 3). (**C**) RNA partition efficiency into condensates, assessed by native gel electrophoresis quantification of supernatant fractions (top panel; n = 3). (**D**) Transmission electron microscopy (TEM) images of selected samples from (**A**) incubated for 48 h. The zoomed inset (orange frame) for the 0-10 condition highlights gel-like globules within condensates and bundled fibrils at the condensate edges. (**E**) Representative FRAP (fluorescence recovery after photobleaching) images for droplets and starbursts (A1PrD-A647 and RNA-A488; condition 400-10). Right panels show FRAP recovery plots with average fluorescence intensity (symbols) and standard deviation (shaded areas) for A1PrD-A647 (purple) and RNA-A488 (green) across different conditions (400-0, 0-10, and 400-10) and aging times. Representative FRAP images are also shown in Figure S7. Sample sizes: 400-0: droplets n = 7, early starbursts n = 3, late starbursts n = 3; 400-10: droplets n = 9, starbursts n = 3; 0-10: droplets n = 4, solid gels n = 5. Darker symbols and shaded areas represent longer-aged samples. Statistical significance determined by two-sided paired Student’s *t*-tests; ns = not significant, with * *p* < 0.05, ** *p* < 0.01, *** *p* < 0.005, and **** *p* < 0.001.

To characterize microenvironmental differences within condensates, we employed fluorescence lifetime imaging microscopy (FLIM), a robust technique for detecting dynamic changes in local environments associated with macromolecular condensation [36,37,38] and aggregation [39] (Figures S3–S5). Longer fluorescence lifetimes typically reflect greater molecular mobility and a more fluid environment, while shorter lifetimes suggest quenching due to increased molecular interactions, crowding, or the formation of dense structures [36,37,38,39,40,41]. Under dilute, non-LLPS conditions (~100 nM A1PrD-A488 without excess unlabeled protein), we observed marked decreases in fluorescence lifetimes upon RNA addition. Lifetimes decreased from ~3.5 ns in the absence of RNA (conditions 0-0 and 400-0) to ~3.2 ns at 400-10, and most dramatically to ~1.5 ns in the 0-10 condition (no salt, high RNA), indicating strong heterotypic A1PrD-RNA interactions (Figure 2A, top row). These effects were partially mitigated by the presence of salt, which likely screens electrostatic interactions. LLPS was induced by adding 20 µM unlabeled A1PrD, resulting in droplet formation under all conditions except 0-0. FLIM images were collected at early (<2 h) and late (>5–24 h) timepoints to monitor the evolution from liquid droplets to starbursts and aggregates. Condensed-phase droplets formed within 2 h exhibited distinct lifetime shifts relative to dilute conditions. Most notably, lifetimes further decreased to ~2.2–2.8 ns for all conditions except 0-10, where lifetime increased to ~2.7 ns, possibly due to buffering of RNA interactions by excess protein (Figure 2A, middle row). These trends were consistent with partitioning data: higher LLPS propensity (Figure 2A) correlated with greater protein and RNA enrichment within condensates (Figure 2B,C). In the 0-0 condition (<2 h), the moderate lifetime reduction may reflect the presence of nano-oligomers or early clustering not visible by microscopy [25,42]. At later stages (>5–24 h; Figure 2A, bottom row), fluorescence lifetimes remained largely unchanged except in the 0-0 condition, which showed significantly reduced lifetimes (~1.5 ns) and the appearance of fibrillar aggregates. TEM analysis corroborated these findings, revealing fine, thin filaments in the 0-0 condition and thicker, bundled fibrils in high-LLPS conditions (Figure 2D). Additionally, we observed ~100 nm spherical nanocondensates coating many of the fibrils. Interestingly, the 0-10 condition, which lacked starburst formation, contained condensates with small, globular gel-like inclusions (see Figure 2A, orange frame). We hypothesize that significant gelation occurs during the aging of condensed droplets. This was validated by fluorescence recovery after photobleaching (FRAP) experiments across timepoints: early-stage droplets showed rapid recovery, while aged gels and starbursts exhibited slowed or absent recovery, indicative of a transition to an arrested or solid-like state (Figure 2E and Figure S7). Altogether, our FLIM data clearly highlight distinct solution microenvironments among different condensate populations. Specifically, these differences align consistently with pathological condensates characterized by homotypic interactions and physiological condensates involving heterotypic interactions with RNA. This distinction underscores the critical role of molecular composition and interaction dynamics in determining the functional and pathological outcomes of biomolecular condensates.

### 3.1. A1PrD Droplet-to-Starburst Morphogenesis in the Presence of RNA

**Figure 3:**
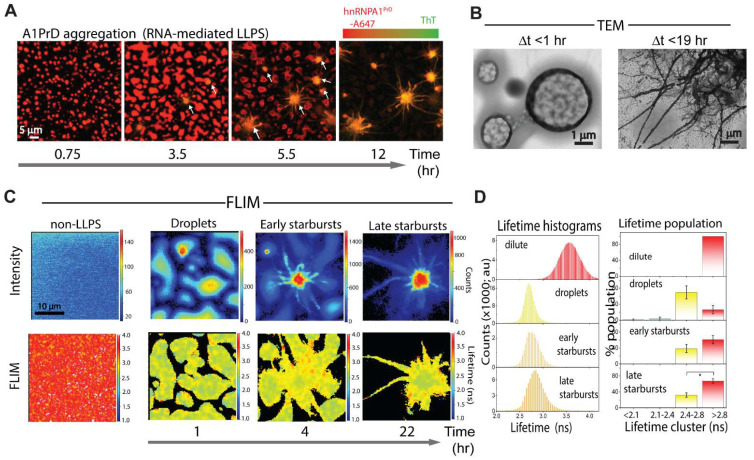
RNA-facilitated aging of A1PrD droplets yields dynamic, fluid starbursts. (**A**) Time-dependent initiation and maturation of A1PrD starbursts monitored by confocal fluorescence microscopy (20 µM A1PrD, 10 µM RNA, ~70 nM A1PrD-A647 (red), and 3 µM thioflavin T (ThT, green) in αβγ buffer containing 200 mM NaCl). (**B**) TEM images depicting an early-stage condensate (1 h) and a mature starburst (19 h). (**C**) Representative confocal (top) and FLIM (bottom) images showing different stages of A1PrD droplet maturation and starburst formation. Conditions include non-LLPS dilute controls and LLPS conditions at 1, 4, and 22 h post initiation. (**D**) Fluorescence lifetime histograms corresponding to the images shown in (**C**). Images were categorized by incubation time and morphological state: surface-wetted droplets (~1 h, n = 4 images), early starbursts (<5 h, n = 7 images), and late starbursts (>12 h, n = 5 images). Right panels present bar graphs showing lifetime distributions clustered into four bins: <2.1 ns (blue), 2.1–2.45 ns (blue-green), 2.45–2.8 ns (yellow), and >2.8 ns (red). Error bars represent the standard deviation (SD) across multiple images (n indicated above). Statistical significance determined by two-sided paired Student’s *t*-tests; with * *p* < 0.05.

To elucidate the mechanism of starburst formation, we employed 4D confocal fluorescence microscopy (timelapse 3D z-stacks) to monitor the transformation of A1PrD liquid droplets into starburst aggregates. We focused on conditions known to robustly promote starburst formation—specifically, 10 µM RNA and 200 mM NaCl. To visualize amyloid formation, Thioflavin T (ThT), a fluorescent dye specific for β-sheet-rich fibrils, was added to the imaging solution. Immediately following LLPS initiation, A1PrD-A647 droplets formed rapidly, exhibiting hallmark liquid behaviors such as fusion and surface wetting within the first hour (Figure 3A and Figure S8). Within a few hours, we observed the emergence of ThT-positive condensates (orange signal; Figure 3A and Figure S8, white arrows), which we interpret as amyloid seed nuclei. These seeds progressively enlarged and developed filamentous protrusions over time (Figure 3A and Figure S8). By 12 h, the majority of condensates had matured into starbursts (Figure 3A, Movie S3). Early-stage protrusions (<3 h) ranged from 0.5 to 1.2 µm in length, while later-stage structures (>5 h) extended to ~5–17 µm. Transmission electron microscopy (TEM) of early droplets revealed a network of fine fibrillar structures (Figure 3B). Consistent with our earlier observations (Figure 2E; 400-10 RNA condition), TEM of mature starbursts revealed a chaotic meshwork of thick, coarse filaments (~200 nm diameter) decorated with nanometer-sized beads or nanocondensates (Figure 3B). These adsorbed nanocondensates may represent early-stage fibrillar precursors or serve as secondary nucleation sites that facilitate fibril elongation or fragmentation, contributing to the intricate and dynamic architecture of starburst morphologies. Consistent with Figure 2A, FLIM analysis revealed no dramatic shift in average fluorescence lifetimes between liquid droplets and starbursts, suggesting that the overall microenvironment within condensates remains relatively stable during morphological evolution (Figure 3C). However, we detected a broadening in the lifetime distribution and a modest shift in the central peak from ~2.6 to ~2.9 ns, which was counterintuitive (Figure 3D). To enable quantitative analysis across multiple datasets, we binned lifetime values into four categories: <2.1 ns, 2.1–2.45 ns, 2.45–2.8 ns, and >2.8 ns (Figure 3D, right panel). Corresponding phasor plots were generated to map spatial localization of lifetime clusters (Figures S9 and S10). Intriguingly, higher lifetime populations predominantly localized to the starburst periphery, which we hypothesize represent newly adsorbed nanocondensates from the dilute phase, structures also observed in TEM analysis (Figure 3B).

**Figure 4:**
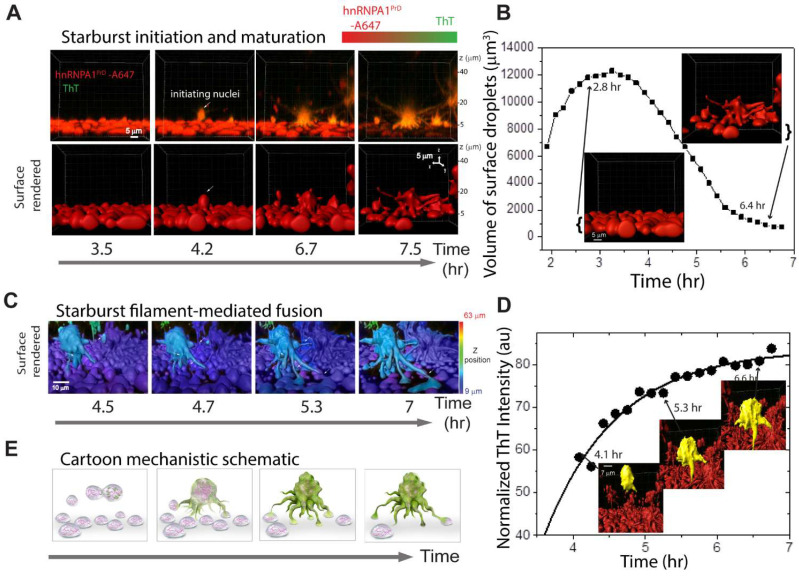
A1PrD starbursts siphon from and infect young condensates. (**A**) Time-dependent maturation of starburst aggregates of 20 µM A1PrD in the presence of 10 µM RNA and 200 mM NaCl (with ~70 nM A1PrD-A647 and 3 µM ThT in αβγ buffer), visualized by 4D confocal microscopy (timelapse 3D z-stack). Additional images provided in Figure S8 and Movies S4 and S5. Bottom panels: Surface-rendered starburst images at various maturation stages generated using Imaris software (Movie S6). (**B**) A quantitative plot of the volume of surface-rendered droplets over time. Representative surface-rendered images at selected timepoints are displayed. (**C**) Surface-rendered visualization depicting fusion events and subsequent in-phase siphoning between starburst filaments and nearby droplets (Movie S7). The primary starburst is highlighted in cyan for clarity. (**D**) A temporal plot of normalized ThT fluorescence intensity from tracked starburst aggregates. Representative surface-rendered images (yellow) at specific timepoints are shown. Similar analyses were performed for five maturing starbursts (Figure S7). (**E**) A schematic illustration of the infection mechanism, wherein ThT-positive (amyloid-rich, green) starburst aggregate siphons material from and recruits ThT-negative condensates.

To further investigate starburst growth dynamics, we tracked individual structures over time and performed morphometric quantification using surface rendering in Imaris ver. 9.2. We observed that initial starburst nuclei often emerged from above the wetted surface (Figure 4A, white arrow). As starbursts expanded and filaments elongated, a concurrent depletion of surface-bound liquid droplets was noted (Figure 4A,B, Movies S4–S6). Quantitative correlation analysis revealed that increasing ThT fluorescence intensity closely paralleled starburst maturation, including increased surface roughness and filament extension (Figure 4C–E; Movies S4–S6; Figures S11–S13). Importantly, early-stage starbursts retained fluid properties, as evidenced by their ability to fuse with other droplets or starbursts. We also observed condensation or shrinkage of some droplets, likely reflecting gelation or adsorption into starburst filaments. These findings align with FLIM data showing heterogeneous lifetime distributions across starburst structures, supporting a model in which each starburst is a multi-nucleated assembly formed through successive fusion and maturation events (Figure 3C, Figures S9 and S10).

Upon closer analysis, we observed that the loss of surface-bound droplets was accelerated by a process resembling ‘in-phase siphoning,’ whereby dynamic filamentous extensions of starbursts actively recruited nearby droplets into the growing structure (Figure 4B; Movie S6). Notably, ThT-positive, amyloid-rich starbursts were capable of recruiting ThT-negative liquid droplets. This observation led us to hypothesize that starbursts exhibit a form of ‘prion-like’ seeding behavior, wherein ThT-positive condensates can convert or ‘infect’ ThT-negative droplets upon contact (Figure 4E and Figure S12; Movie S7).

Interestingly, we observed similar starburst morphogenesis in the absence of RNA (Movie S8), although fewer LLPS droplets were formed under these conditions. As a result, fusion events between droplets and starbursts were reduced, and the resulting starbursts had smaller core diameters compared to RNA-rich conditions (compare 400-0 and 400-10 in Figure 2A). These data suggest that filamentation primarily propagates outward from the interior of starbursts. However, we also documented cases where filamentous protrusions appeared to originate from the condensate interface, particularly at the boundary of arrested or gel-like condensates (Figure S14, Movie S8), consistent with prior descriptions of interface-driven surface nucleation [18,23]. We propose that the capacity of starbursts to undergo both filament elongation and droplet fusion depends on their retention of a fluid and dynamic internal environment during early stages. This is supported by FRAP experiments, which demonstrated rapid fluorescence recovery in early-stage starbursts (Figure 2E and Figure S7), confirming their liquid-like character and compatibility with dynamic remodeling and recruitment processes.

### 3.2. Starburst Evolution of A1PrD in the Presence of a Crowding Agent

**Figure 5:**
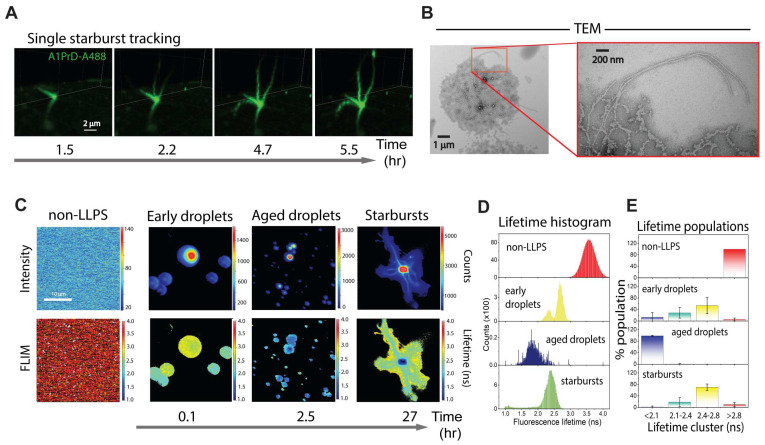
A1PrD droplet aging and starburst formation in the presence of the crowding agent (PEG). PEG-enhanced protein condensation was monitored using 20 µM unlabeled A1PrD, ~70 nM A1PrD-A488, 10% *w/v* PEG-8K, and 200 mM NaCl in αβγ buffer. (**A**) Time-dependent initiation, maturation, and fibrillar extension of A1PrD starbursts visualized by 4D confocal microscopy (timelapse 3D z-stack). (**B**) A TEM image of an aged condensate showing fibrillar extensions. (**C**) Representative confocal (top row) and FLIM (bottom row) images at different stages of droplet maturation and starburst formation: non-LLPS dilute controls and LLPS conditions at ~5 min, 2.5 h, and 27 h post LLPS initiation. (**D**) Fluorescence lifetime histograms corresponding to the images shown in (**C**). (**E**) Lifetime data grouped according to incubation time and morphological states: early droplets (10–30 min, n = 3 images, >200 droplets); aged droplets (2–3 h, n = 4 images, >200 droplets); and mature starbursts (n = 7 images). Lifetimes were clustered into four bins: <2.1 ns (blue), 2.1–2.45 ns (blue-green), 2.45–2.8 ns (yellow), and >2.8 ns (red). Error bars represent the average and SD from multiple images (n indicated).

Crowding agents such as polyethylene glycol (PEG) are widely used to promote LLPS [11,12]. To compare the mechanisms underlying NaCl-/RNA-facilitated versus crowding agent-mediated starburst formation, we performed timelapse confocal microscopy tracking under conditions containing 10% (*v*/*v*) PEG-8K. PEG-induced LLPS yielded sparser droplets, enabling the tracking of individual starburst evolution events (Figure 5A and Figure S15, Movie S10). The morphogenesis from droplet to starburst closely resembled that previously described for FUS [11], characterized by thickening and elongation of radial filaments. TEM analysis of early condensates revealed the presence of oligomeric intermediates and nascent fibrils that initiated from within the condensates and extended outward (Figure 5B). FLIM analysis revealed distinct differences in fluorescence lifetime patterns compared to RNA-mediated condensates (Figure 5C–E). As expected, dilute A1PrD solutions (~70 nM A1PrD-A488) exhibited high lifetimes (~3.5 ns; Figure 5C–E; phasor plots in Figures S16–S18). Applying the same binning strategy for lifetime clusters (<2.1, 2.1–2.45, 2.45–2.8, >2.8 ns), early PEG-induced droplets (~30 min) displayed at least two dominant lifetime populations (~2.2 and ~2.5 ns; Figure 5D,E). Notably, fusion events were observed between droplets with distinct lifetime signatures (Figure 5C, early droplets panels), indicating heterogeneity in their internal environments. With continued incubation, droplets matured into structures exhibiting lifetimes <2 ns (Figure 5C–E), with some transitioning into starbursts (Figure 5C and Figure S15). As seen in RNA-induced starbursts, the filamentous extensions and peripheral shell of PEG-induced starbursts retained longer lifetimes (~2.2–2.5 ns), whereas their dense core exhibited shorter lifetimes (<2 ns), suggestive of increased local quenching due to denser molecular packing (Figure 5C–E and Figures S16–S18). A gradient in lifetimes from the outer interface to the center core was consistently observed, reflecting spatial variation in local microenvironments. Given the high unlabeled-to-labeled protein ratio (~300:1) and uniform fluorescence intensity across droplets, the observed lifetime reductions likely indicate increased fluorophore quenching driven by molecular crowding in the core. The gradual time-dependent decrease in lifetimes and the emergence of spatial gradients are consistent with prior studies suggesting that condensates behave as aging Maxwell fluids [43], transitioning gradually from liquid-like to gel-like or solid-like states rather than undergoing abrupt phase changes. We speculate that the longer lifetimes at droplet and starburst interfaces result from dynamic exchange with the surrounding dilute phase, which averages local environments over time. Furthermore, these interfaces may function as scaffolds or ‘magnets’ for ongoing nucleation and fibril elongation, as similarly observed in RNA-induced starbursts. Altogether, PEG-mediated starburst formation appears to involve three key features: [1] fibrillation originating internally and propagating outward, [2] droplet aging via gelation or glass-like dynamics, and [3] secondary fibril nucleation and branching at interfaces and filament tips.

### 3.3. LLPS vs. Non-LLPS Aggregation Mechanisms

**Figure 6:**
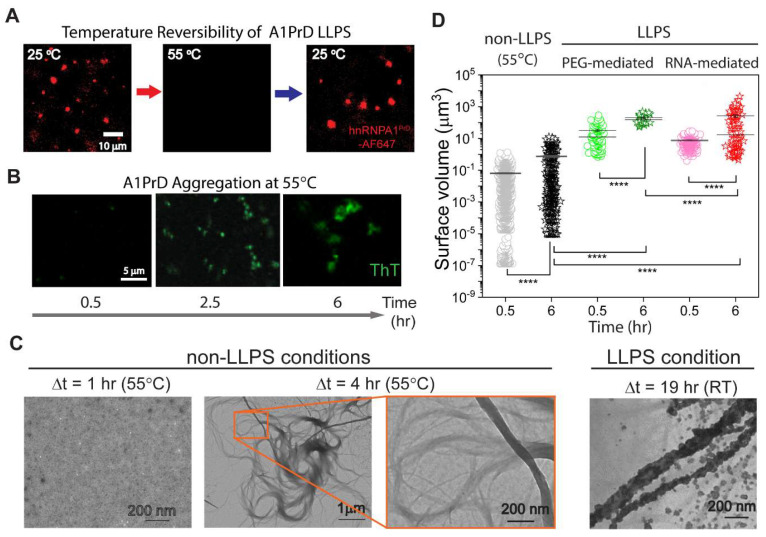
Morphological distinctions between aggregates formed under non-LLPS and LLPS conditions. (**A**) Fluorescence microscopy data demonstrating temperature-dependent reversibility of RNA-mediated A1PrD LLPS droplets (10 µM RNA, 20 µM A1PrD, ~70 nM A1PrD-A647, and 3 µM ThT in αβγ buffer). (**B**) Confocal fluorescence images showing A1PrD aggregates formed at elevated temperature (55 °C) under identical solution conditions as described in (**A**). (**C**) TEM images illustrating A1PrD particulate and fibrillar aggregates obtained by incubating samples (10 µM RNA, and 20 µM A1PrD in αβγ buffer) at 55 °C for 1 h (particles) and 4 h (fibrils). (**D**) Comparative size distributions of particle aggregates formed under non-LLPS versus LLPS conditions, facilitated by either PEG or RNA, at incubation times of 30 min or 6 h. Statistical significance determined by two-sided paired Student’s *t*-tests; with **** *p* < 0.001.

Traditional protein aggregation studies are typically conducted under dilute conditions and proceed via mechanisms involving protein misfolding or the nucleation of fibrillation-competent species [44]. To investigate A1PrD aggregation under non-LLPS conditions, we employed temperature modulation to reverse LLPS and bypass phase separation-mediated pathways [45,46]. We emphasize that this elevated-temperature experiment was intended as a non-LLPS reference pathway, not as a direct kinetic surrogate for the room-temperature aging of LLPS droplets. A1PrD condensates formed via RNA-mediated LLPS are reversible upon temperature elevation or treatment with the chemical disruptor 1,6-hexanediol [12,18,47]. In contrast, aged starbursts remain resistant to such perturbations (Figure 6A, Movie S11, Figure S19). We utilized this property to induce aggregation via a non-LLPS route by raising the temperature to 55 °C, which led to rapid droplet dissolution and allowed us to observe A1PrD aggregation from the dilute phase. Within 30 min, we detected small, irregularly shaped aggregates by fluorescence microscopy and TEM (Figure 6B,C). These microscopic aggregates likely originate from oligomeric intermediates or cluster-like nuclei, which gradually grow into larger irregular structures over several hours. However, even after prolonged incubation, non-LLPS aggregates remained markedly smaller than those formed via LLPS pathways (e.g., PEG- and RNA-mediated conditions; Figure 6D). TEM analysis revealed that non-LLPS aggregates were composed of fine, thin filaments (Figure 6C), resembling those observed under low-salt and RNA-free conditions (0-0; Figure 2E). In contrast, LLPS-mediated aggregates were composed of thicker, coarser filaments (Figure 6C), underscoring a mechanistic divergence between aggregation pathways. Together with the 0-0 and 400-0 conditions described above, these data indicate that A1PrD can aggregate without PEG or RNA, but the non-LLPS pathway is slower and yields morphologically distinct, finer fibrillar assemblies. These findings highlight the distinct morphologies and assembly dynamics that result from LLPS-mediated versus non-LLPS aggregation processes.

### 3.4. Interplay Between LLPS, Gelation, and Fibrillation Governs Condensate Morphogenesis

**Figure 7:**
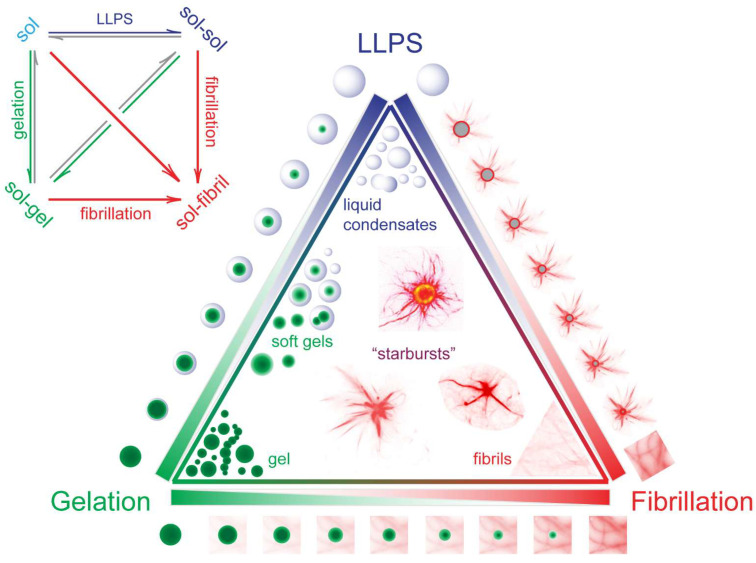
Morphological complexity driven by phase behavior and transitions. The interplay between liquid-liquid phase separation (LLPS), gelation, and fibrillation defines the properties of biomolecular condensates. LLPS mediates transitions between single-phase solutions (sol) and multiphase states (sol-sol). Gelation converts these states into sol-gel networks, while fibrillation emerges from either solution or gel states. For A1PrD, LLPS and gelation processes are largely reversible, whereas fibrillation is irreversible. Depending on the predominant process and the specific phase transitions involved, A1PrD condensates adopt diverse morphologies, including liquid droplets, gels, fibrils, and structurally varied ‘starburst’ aggregates.

To unify our findings and provide a conceptual framework for how different physicochemical processes shape condensate morphology, we developed a schematic model depicted in Figure 7. This model illustrates how the relative kinetics of LLPS, gelation, and fibrillation govern the resulting aggregate structures of A1PrD and similar prion-like proteins. Our data indicate that gelation can either delay fibrillation or promote it, depending on when arrest occurs relative to nucleation. If gelation outpaces fibrillation, arrested or gel-like condensates emerge, as observed for A1PrD in the presence of RNA, and these states show limited internal restructuring and reduced amyloid accumulation. In contrast, when LLPS and fibrillation proceed more rapidly than full arrest, large starbursts form with prominent filamentous protrusions and expanded core diameters, indicative of continued internal fluidity that facilitates outward fibril growth. When partial gelation follows initial LLPS, heterogeneous condensates and interfaces can also promote nucleation, yielding more solid-like droplets with small dense cores and interface-localized filamentation. In this framework, starbursts are best interpreted as metastable mixed states that arise after LLPS but before complete conversion to more fibril-dominant assemblies. The transition map is also modulated by the solution variables identified here: RNA generally shifts the system toward heterotypic, buffered condensates; elevated salt or PEG favors homotypic condensation and starburst growth; and elevated temperature suppresses LLPS and reveals a distinct non-LLPS route. Together, these scenarios illustrate how the timing and interplay of LLPS, gelation, and fibrillation determine the material properties, internal architecture, and morphological diversity of protein condensates. Understanding these kinetic relationships is crucial for deciphering pathological aggregation pathways and may inform strategies for modulating condensate behavior in disease contexts.

## 4. Discussion

The data presented here show that A1PrD morphology is dictated by a kinetic competition among LLPS, gelation, and fibrillation rather than by LLPS alone [21,22,23,34,43]. RNA-rich conditions favor heterotypic interactions and highly dynamic condensates with relatively low SDS-insoluble material, whereas salt-rich or crowding conditions shift condensates toward homotypic interactions, starburst growth, and amyloid accumulation. In this framework, gelation can either delay fibrillation or promote it, depending on timing: early arrest in RNA-rich droplets buffers protein and suppresses fibril growth, whereas partial aging after LLPS creates heterogeneous cores and interfaces that support nucleation and outward filament extension. The starburst morphology is therefore best viewed as a metastable intermediate that couples liquid-like, gel-like, and fibrillar features.

Because this study isolates A1PrD, it resolves the intrinsic behavior of the low-complexity domain but does not capture the RNA selectivity imparted by the RRM domains or the full regulatory environment of cells. In vivo, molecular crowding, compartment-specific ionic conditions, ATP-dependent remodeling, chaperones, post-translational modifications, and selective RNA binding are all expected to shift the phase boundaries and kinetic balances defined here [8,12,21,22,23,34]. Even so, the present results likely capture a general physical principle relevant to other disease-linked prion-like proteins, including FUS and TDP-43: sequence-encoded sticker-spacer composition and charge patterning determine how condensates age, while heterotypic ligands can buffer or redirect that trajectory.

An important implication is that condensates need not be immediately fibrillar to become pathogenic. Arrested gels and starbursts may sequester proteins and RNA, reduce bioavailability, and generate loss-of-function states even before conversion to mature amyloid [21,43]. Future work with full-length hnRNPA1 or RRM-containing constructs, defined RNAs, and more cell-like environments should clarify how sequence context and molecular partners tune the transition between physiological condensates and pathological inclusions.

## 5. Conclusions

In conclusion, A1PrD condensate morphology is not determined by LLPS alone but by the relative rates of LLPS, gelation, and fibrillation under defined solution conditions. RNA-rich conditions buffer aggregation and favor more dynamic condensates, whereas salt- or PEG-promoted homotypic interactions drive starburst formation, amyloid enrichment, and recruitment of neighboring condensates. These results provide a physical framework for how distinct pathological inclusion morphologies can emerge from the same protein and suggest that tuning condensate material properties may help limit their maturation into pathogenic assemblies.

## Data Availability

The authors declare that the main data supporting the findings of this study are available within the article and its Supplementary Materials. Extra data are available from the corresponding authors upon request.

## Supplementary Materials

The following supporting information can be downloaded at: https://www.mdpi.com/article/10.3390/biom16040492/s1, Figure S1: Dependence of condensate and aggregate sizes on A1PrD concentration; Figure S2: Anti-correlation between A1PrD LLPS potential and SDS-insoluble fibril formation; Figure S3: Fluorescence Lifetime Imaging Microscopy (FLIM) data analysis approaches; Figure S4: Fluorescence lifetimes measured by FLIM are independent of intensity and analysis method; Figure S5: Fluorescence lifetime measurements remain consistent across varying binning times and imaging depths; Figure S6: Fluorescence lifetimes of A1PrD under varying solution conditions; Figure S7: FRAP measurements reveal reduced recovery in aged droplets and starbursts; Figure S8: RNA-mediated aging of A1PrD condensates; Figure S9: RNA-mediated A1PrD droplets mature into fluid and dynamic starbursts; Figure S10: Representative FLIM images of RNA-mediated aging of A1PrD condensates; Figure S11: Representative z-stack FLIM images monitoring RNA-mediated aging of A1PrD condensates; Figure S12: A1PrD starbursts fuse with, siphon material from, and infect young condensates; Figure S13: RNA-mediated aging of A1PrD condensates correlates with increased ThT fluorescence; Figure S14: Formation and filamentation of A1PrD starbursts from solid gel clusters; Figure S15: PEG-mediated aging of A1PrD condensates; Figure S16: Aging and starburst formation of A1PrD droplets in the presence of PEG; Figure S17: Representative FLIM images tracking PEG-mediated A1PrD droplet aging; Figure S18: Representative z-stack FLIM images tracking PEG-mediated aging of A1PrD droplets; Figure S19: Assessment of droplet and starburst reversibility with temperature and hexanediol; Movie S1: SDS-mediated dissolution of A1PrD solid gel condensates; Movie S2: SDS-mediated dissolution of A1PrD starburst cores; Movie S3: Aging dynamics of A1PrD RNA-mediated LLPS and aggregation; Movie S4: Starburst initiation, growth through material sequestration, and condensate infectivity as regulated by fluid-solid balance; Movie S5: Starburst initiation, growth via material sequestration, and condensate infectivity governed by fluid-solid balance; Movie S6: Surface-rendered visualization of starburst initiation, growth by material sequestration, and condensate infectivity; Movie S7: In-phase fusion, siphoning, and infectivity of A1PrD starbursts; Movie S8: Initiation and growth of A1PrD starbursts without RNA; Movie S9: Initiation of filamentous starbursts from A1PrD solid gel clusters; Movie S10: PEG-mediated aging of A1PrD droplets drives filament formation from individual condensates transitioning from liquid to solid phases; Movie S11: Thermal reversibility of A1PrD liquid droplets.
