# Supplementary material for: Modulation of Biomolecular Aggregate Morphology and Condensate Infectivity
Source: Biomolecules. 2026 Mar 25;16(4):492. doi: 10.3390/biom16040492 (PMC13113973; doi:10.3390/biom16040492)

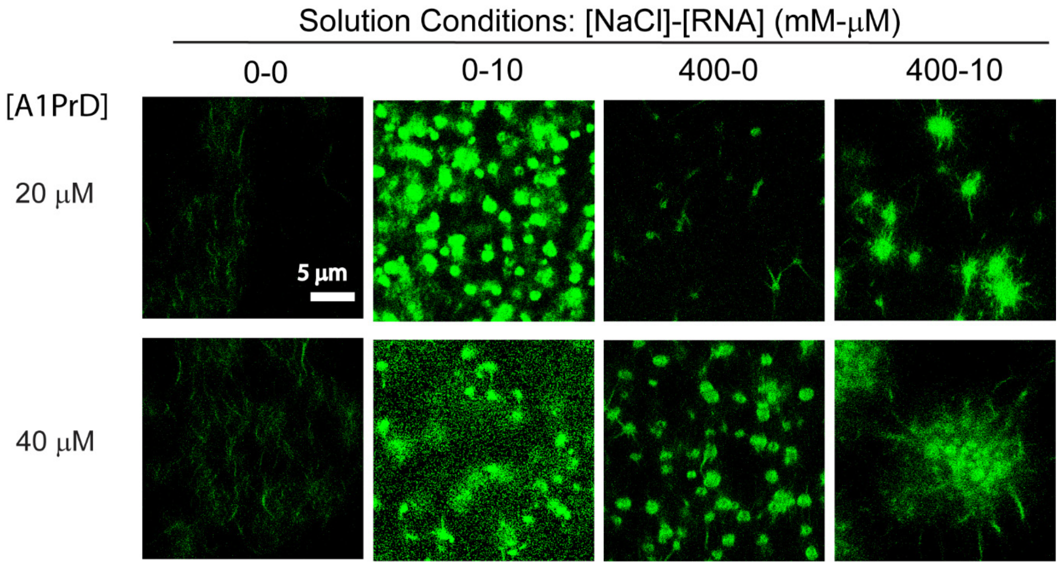

Figure S1. Dependence of condensate and aggregate sizes on A1PrD concentration. Confocal microscopy images of A1PrD (100 nM A1PrD-A488 combined with either 20 or 40 μM unlabeled A1PrD) under varying solution conditions ([NaCl]-[RNA], mM-μM: 0-0, 0-10, 400-0, and 400-10).

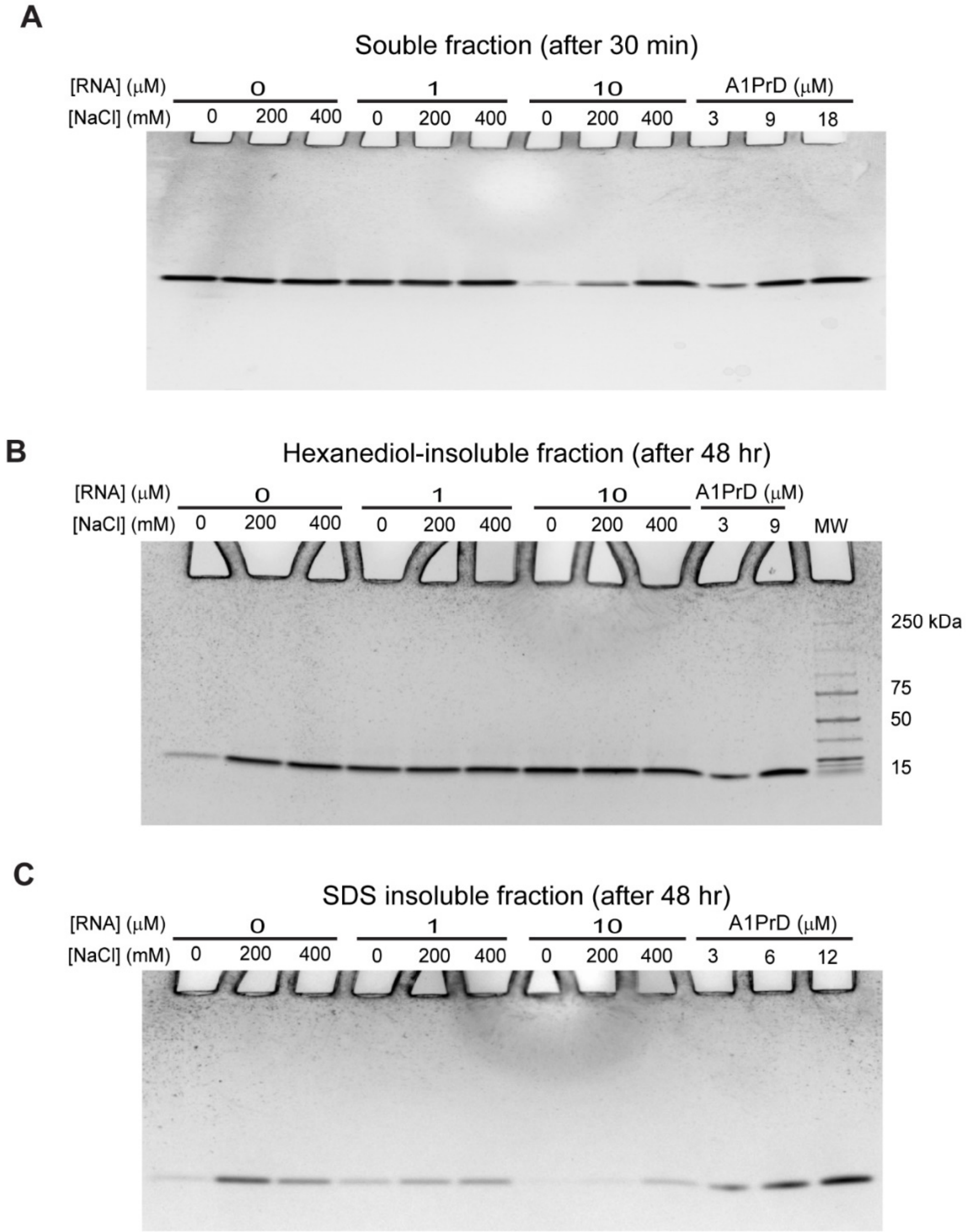

Figure S2. Anti-correlation between A1PrD LLPS potential and SDS-insoluble fibril formation. 20 μM A1PrD samples were prepared under various salt and RNA conditions in αβγ buffer. (A) SDS-PAGE analysis of soluble fractions (supernatants collected after 30 min of LLPS initiation). Similar results were confirmed in three independent experiments. (B) SDS-PAGE analysis of 1,6-hexanediol-insoluble fractions (pellets collected after 48 hr incubation in 20% 1,6-hexanediol). Similar results were confirmed in three independent experiments. (C) SDS-PAGE analysis of SDS-insoluble fractions (pellets collected after 48 hr incubation in 2% SDS). Similar results were confirmed in three independent experiments. Lanes 1-3 represent conditions with 0 μM RNA at increasing salt concentrations (lane 1: 0 mM NaCl; lane 2: 200 mM NaCl; lane 3: 400 mM NaCl). Lanes 4-6 and subsequent lanes represent similar salt gradients with RNA concentrations of 1 or 10 μM. The final three lanes contain 3, 9, and 18 μM A1PrD reference standards for quantification.

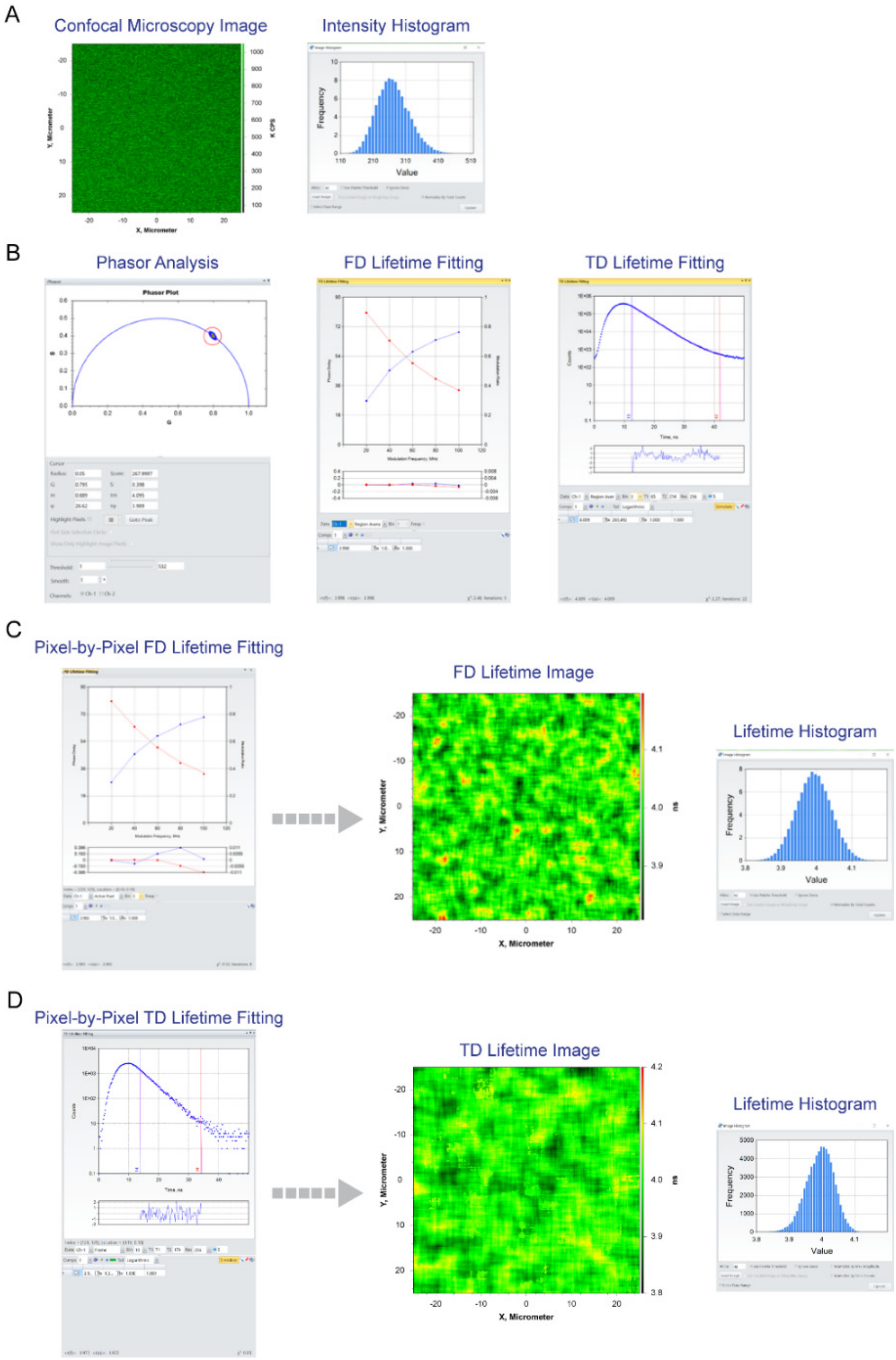

Figure S3. Fluorescence Lifetime Imaging Microscopy (FLIM) data analysis approaches. (A) Spatially resolved fluorescence intensity measurements acquired using the ALBA system by x-y scanning, with 10 nM rhodamine 110 in water serving as a control (fluorescence lifetime = 4.0 ns). The histogram distribution of measured intensities confirms the presence of a single molecular species. (B) Various methodologies are available for FLIM data analysis, including phasor plot analysis, frequency domain (FD) lifetime fitting, and time domain (TD) lifetime fitting. These analyses can be applied using different formats: pixel-by-pixel, selected groups of pixels (spatially or threshold-based), or globally (using all pixels). (C-D) Fluorescence lifetime fitting can be executed pixel-by-pixel across the entire x-y frame, with optional data binning depending on noise levels. Noise is influenced by

sample properties and measurement parameters, including dwell time per pixel, frame dimensions, and total pixel count.

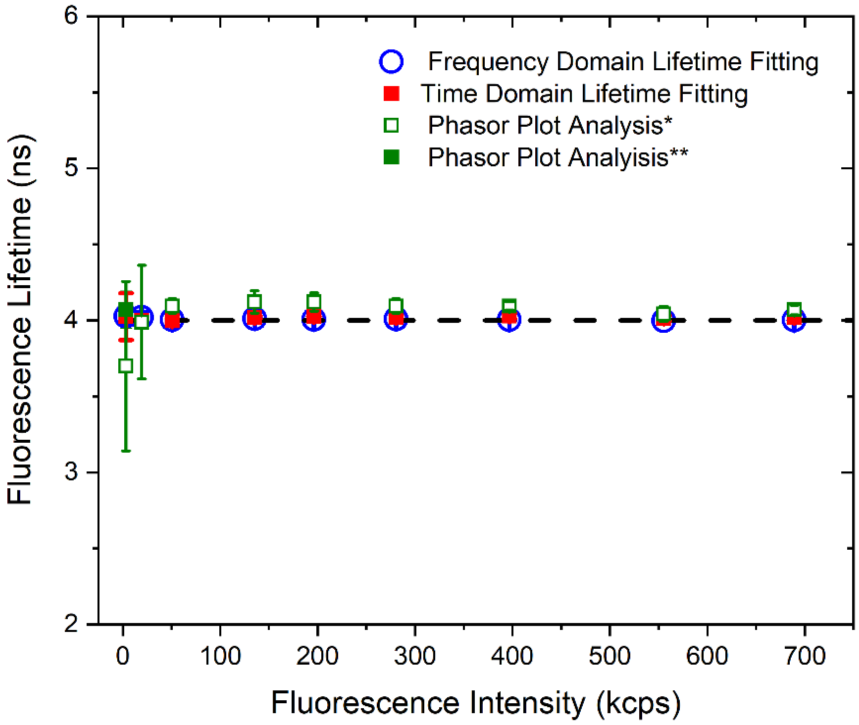

Figure S4. Fluorescence lifetimes measured by FLIM are independent of intensity and analysis method. FLIM data were obtained using 10 nM rhodamine 110 in water as a control, with laser power varying from ~1-100 μW, corresponding to an average fluorescence intensity range of ~3-700 kcps. Note: Instrument shutters positioned before the avalanche photodiode detectors close at intensities above ~800 kcps, and typical experimental settings limit measurements to a maximum intensity of ~500 kcps to maintain linear detector response. Measured lifetimes remain consistent across different analysis methods and signal intensities, except when signals become extremely low (~3 kcps), approaching intensities typical of single-molecule measurements (*). Under such low-signal conditions, reliable lifetime measurements can still be achieved by applying increased Gaussian smoothing (**) to the data.

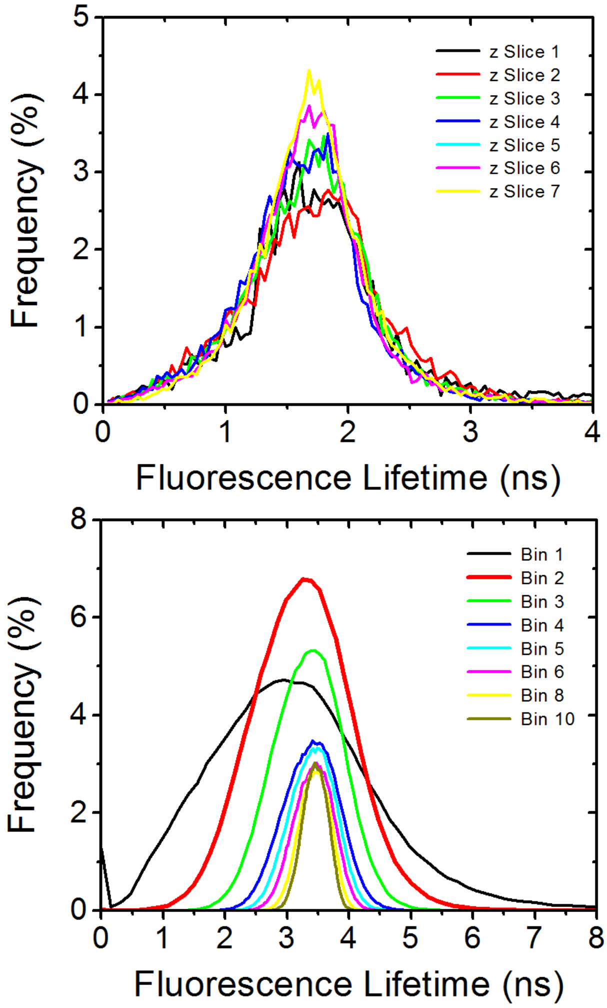

Figure S5. Fluorescence lifetime measurements remain consistent across varying binning times and imaging depths. (Left panel) FLIM lifetime histograms at different binning times. Conditions: ~70 nM A1PrD-A488 in 10% PEG-8K. (Right panel) FLIM lifetime histograms across various z-slices (each 6 μm thick). Conditions: 20 μM A1PrD with ~70 nM A1PrD-A488 in 10% PEG-8K.

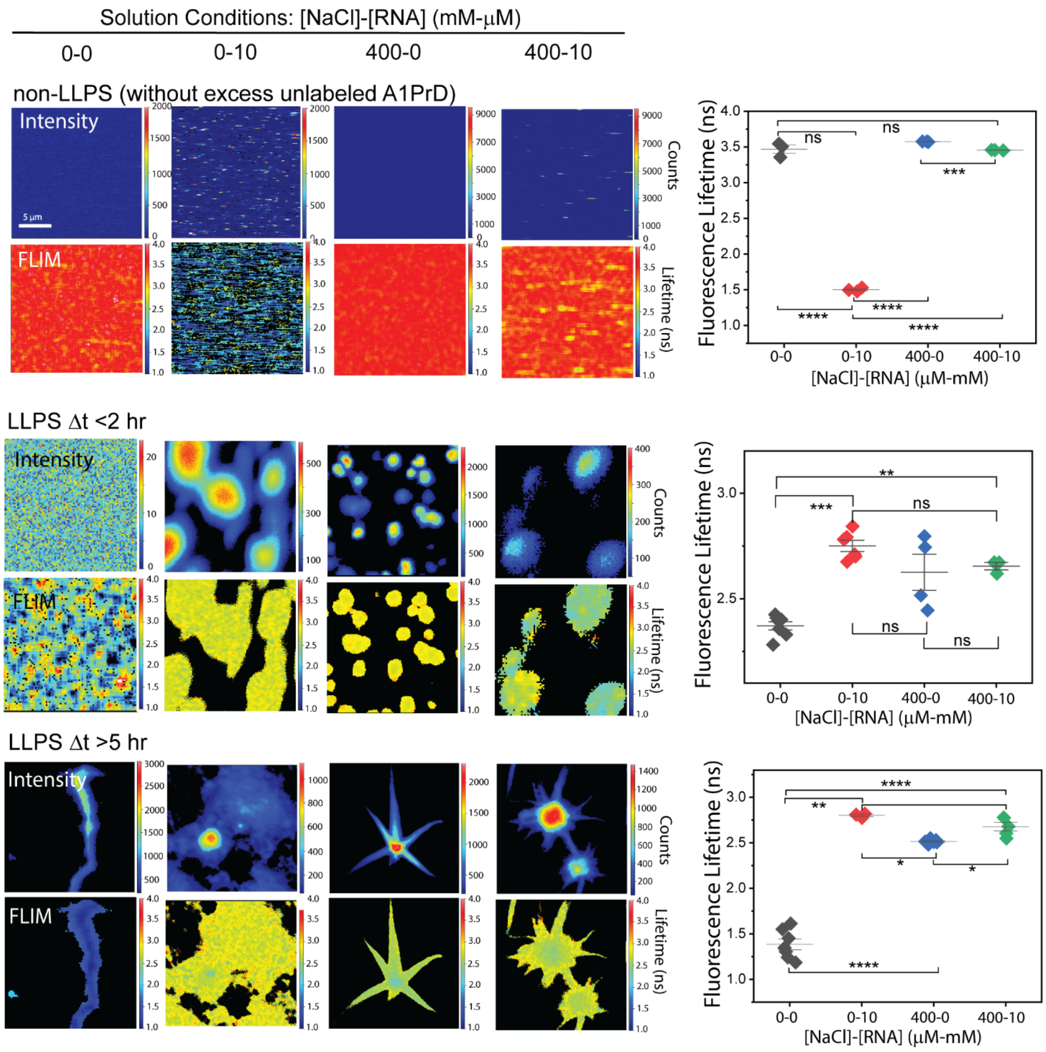

Figure S6. Fluorescence lifetimes of A1PrD under varying solution conditions. (Left, top rows) Confocal fluorescence images illustrating A1PrD starburst maturation at different time points (<2 hr and >5–24 hr post-LLPS initiation). (Left, bottom rows) Corresponding FLIM images. (Right panels) Quantified FLIM lifetimes (mean ± SD; n=3–7 images). Statistical significance determined by two-sided paired Student's t-tests: ns = not significant, *P<0.05, **P<0.01, ***P<0.005, ****P<0.001.

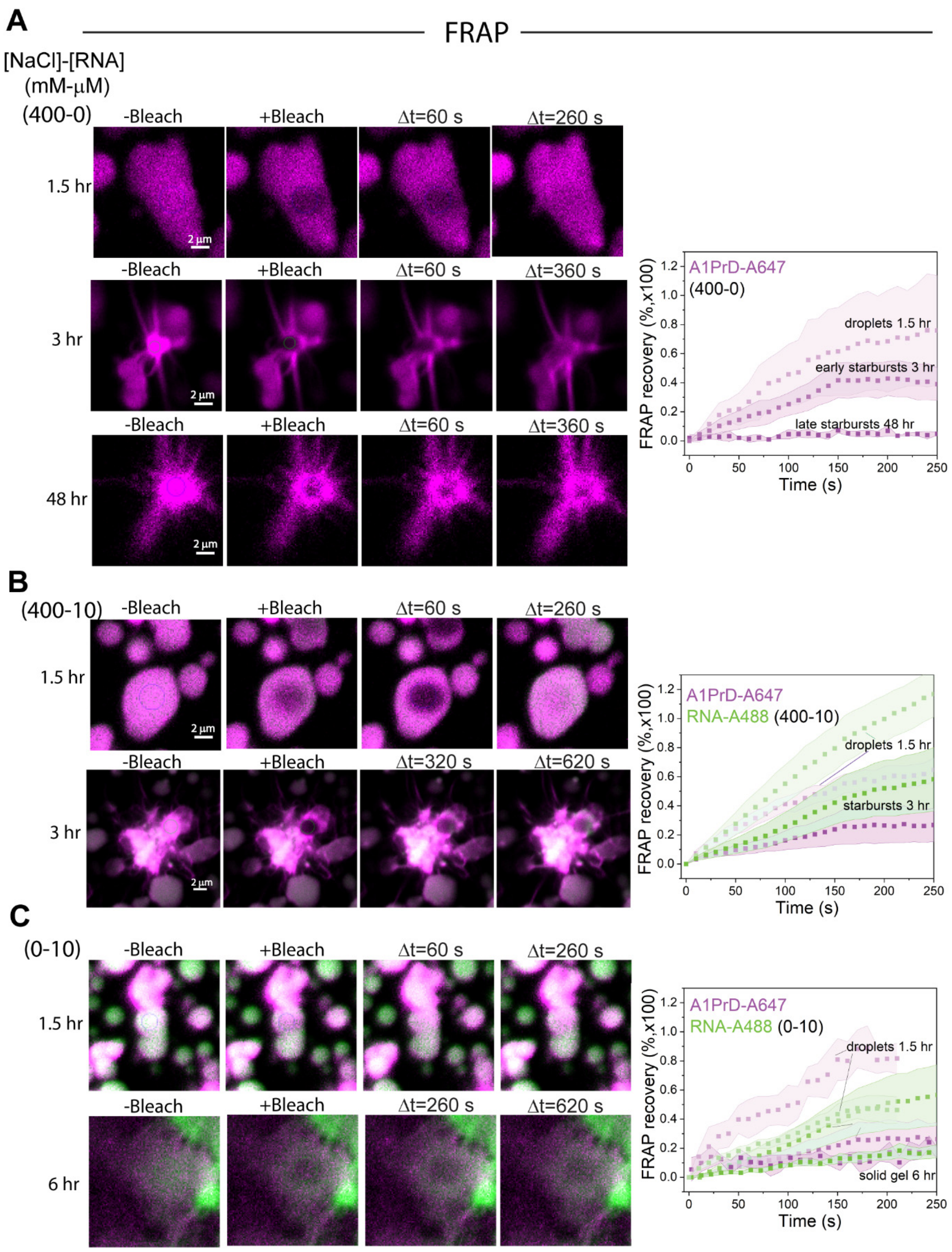

Figure S7. FRAP measurements reveal reduced recovery in aged droplets and starbursts. Representative FRAP images (pre-bleach and post-bleach at specified time intervals) for samples at varying solution conditions ([NaCl]-[RNA], mM-µM: 400-0 (A), 400-10 (B), and 0-10 (C)). Corresponding FRAP recovery plots indicate average fluorescence intensities (symbols) and standard deviations (shaded areas) of A1PrD-A647 (purple) and RNA-A488 (green). Sample sizes: 400-0: droplets (n=7), early starbursts (n=3), late starbursts (n=3); 400-10: droplets (n=9), starbursts (n=3); 0-10: droplets (n=4), solid gels (n=5). Darker symbols and shaded areas represent longer-aged samples.

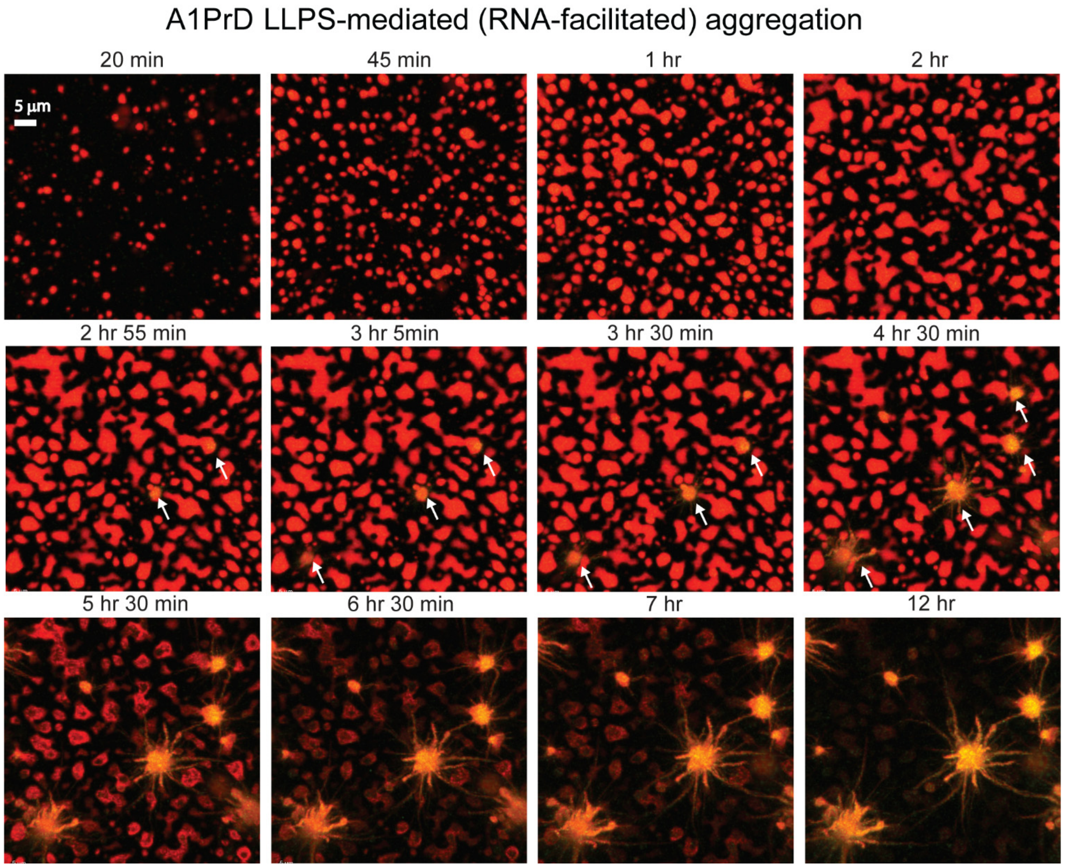

Figure S8. RNA-mediated aging of A1PrD condensates. Time-lapse confocal microscopy images tracking RNA-mediated aging of A1PrD droplets and their subsequent transition to starburst aggregates. Experimental conditions: 20 μM A1PrD, 10 μM RNA, ~70 nM A1PrD-A647 (red), and 3 μM ThT (green) in αβγ buffer. The average droplet diameter was 2.2 ± 0.4 μm with an aspect ratio (long axis divided by short axis) of 1.3 ± 0.3 (n=414 droplets). Aspect ratio values greater than 1 primarily result from droplet fusion events.

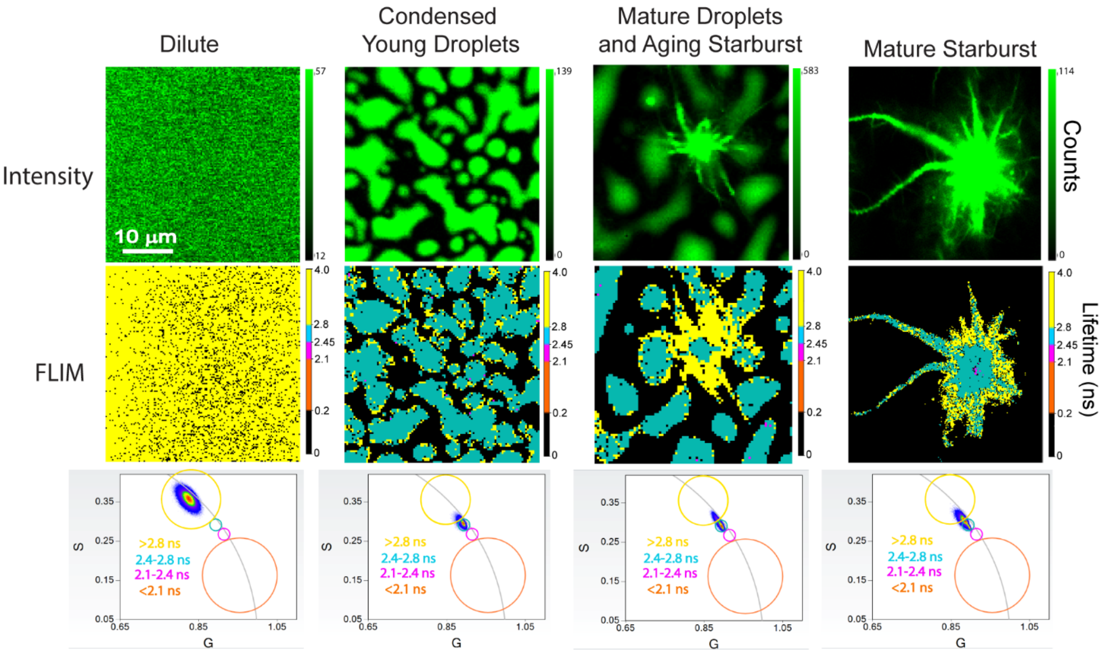

Figure S9. RNA-mediated A1PrD droplets mature into fluid and dynamic starbursts. Confocal microscopy (top row; A1PrD-A488, green) and corresponding FLIM images (middle row) illustrating distinct stages of droplet formation and starburst maturation: non-LLPS dilute conditions, and LLPS conditions at ~1, ~4, and ~22 hr post-LLPS initiation (left to right). Bottom panels show phasor plots with fluorescence lifetimes clustered into four categories: <2.1 ns (orange), 2.1–2.45 ns (magenta), 2.45–2.8 ns (coral blue), and >2.8 ns (yellow). Mean phase lifetimes (τψ) for each cluster are indicated. Data compiled from 35 measurements across two independent replicates.

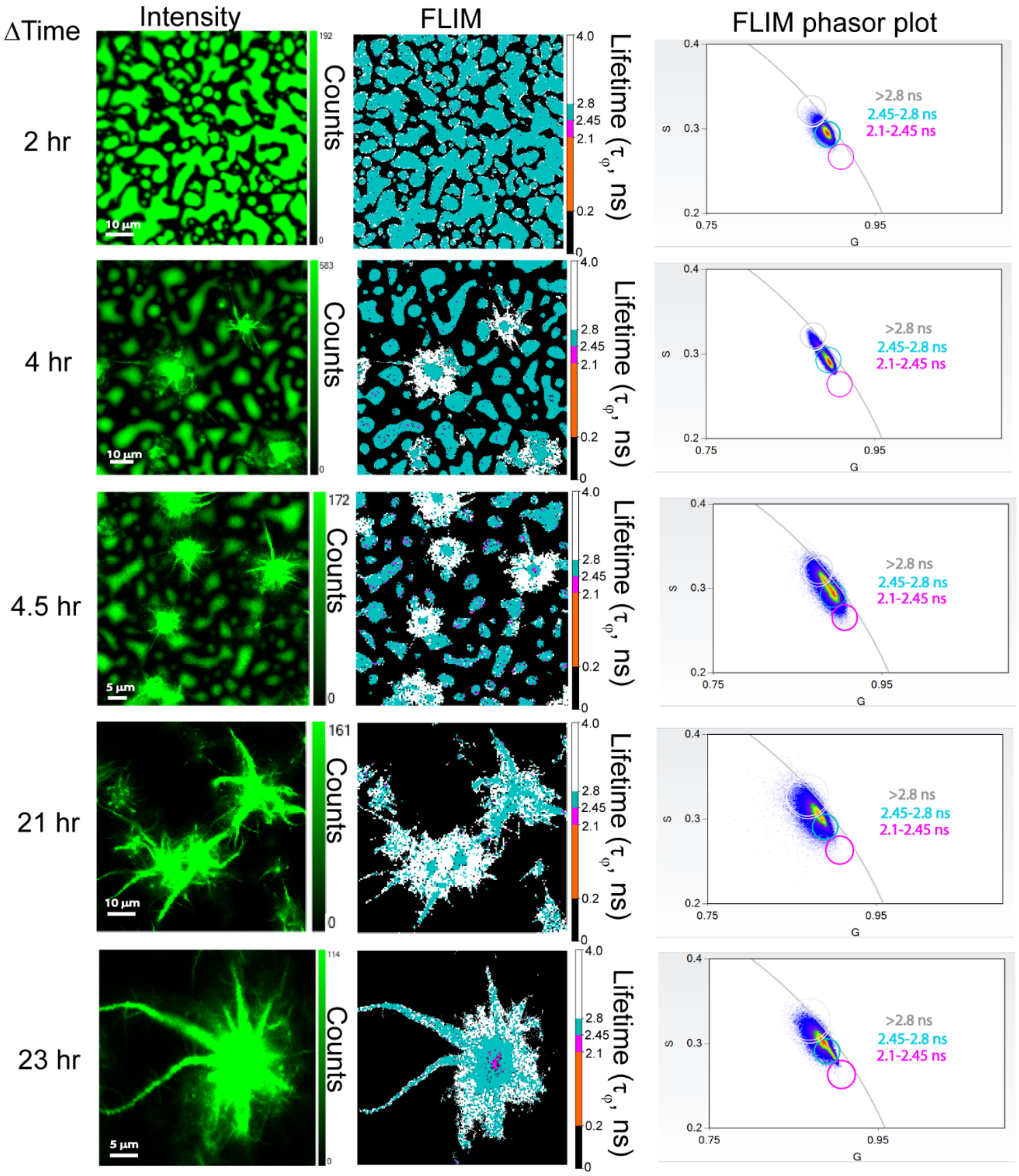

Figure S10. Representative FLIM images of RNA-mediated aging of A1PrD condensates. FLIM experiments performed with 20 µM A1PrD, 10 µM RNA, and ~70 nM A1PrD-A488 in αβγ buffer. Confocal (left panels) and corresponding FLIM images (middle panels) illustrate progressive aging of RNA-mediated A1PrD droplets into starburst aggregates. Right panels show phasor plots with fluorescence phase lifetimes categorized into three clusters: 2.1–2.45 ns (magenta), 2.45–2.8 ns (coral blue), and >2.8 ns (white/gray).

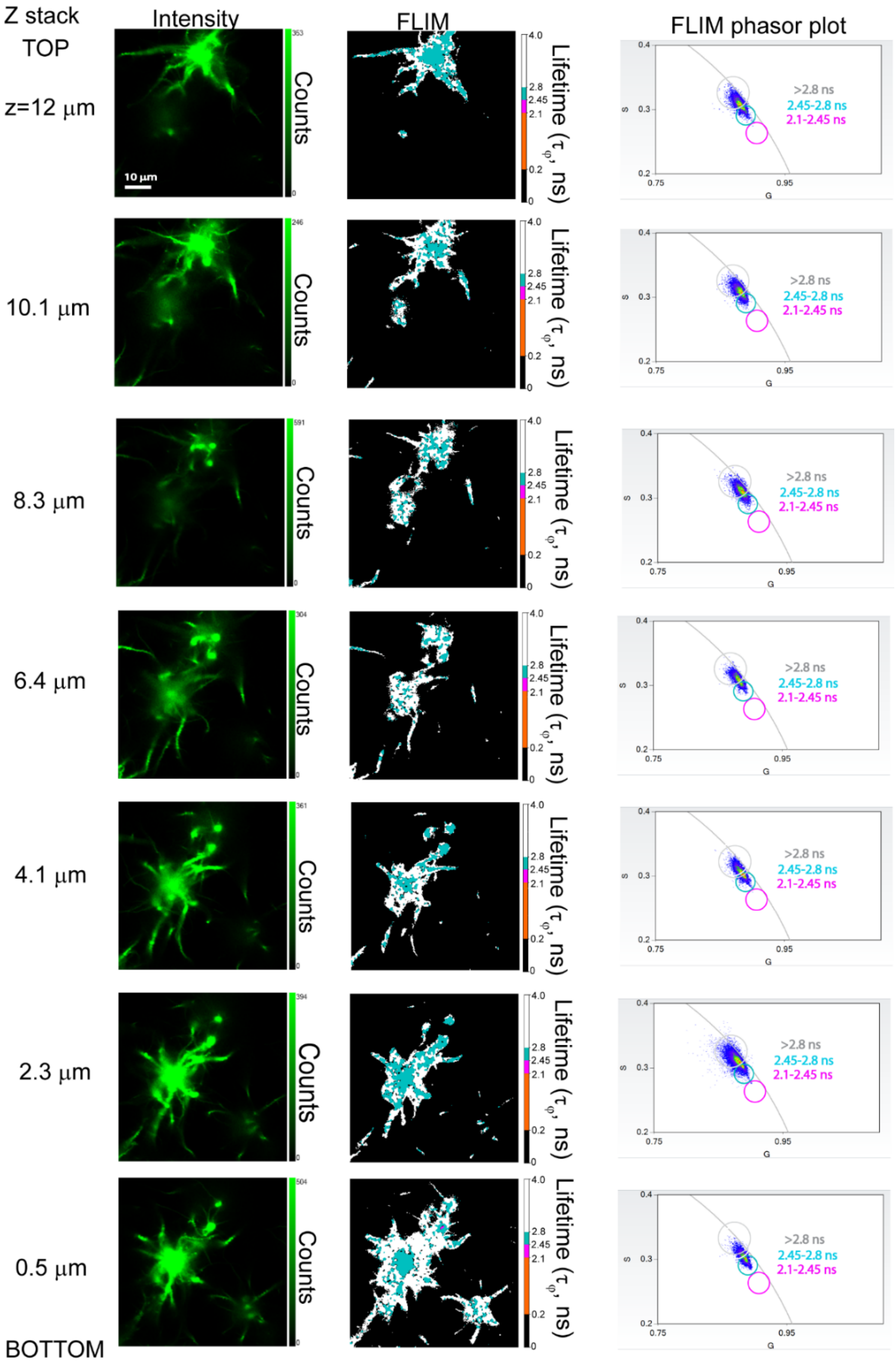

Figure S11. Representative z-stack FLIM images monitoring RNA-mediated aging of A1PrD condensates. Confocal microscopy (left panels) and corresponding FLIM images (middle panels) illustrate A1PrD starburst formation. Phasor plots (right panels) depict fluorescence lifetimes categorized into three clusters: 2.1–2.45 ns (magenta), 2.45–2.8 ns (coral blue), and >2.8 ns (white).

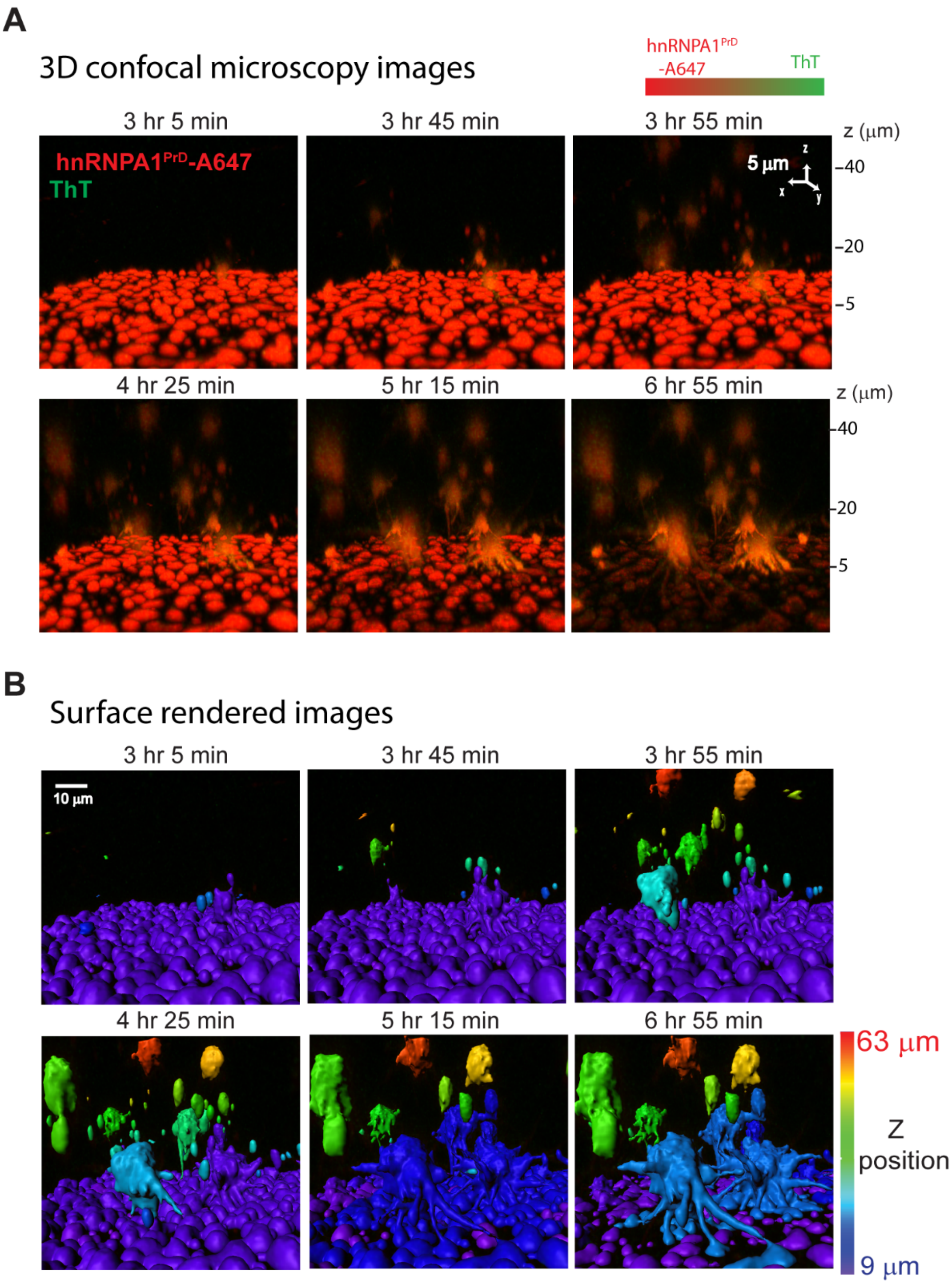

Figure S12. A1PrD starbursts fuse with, siphon material from, and infect young condensates. (A) 4D confocal microscopy (time-lapse 3D z-stack) images tracking RNA-mediated aging of A1PrD droplets and subsequent starburst formation. Conditions: 20 μM A1PrD, 10 μM RNA, ~70 nM A1PrD-A647 (red), and 3 μM ThT (green) in αβγ buffer. (B) Surface-rendered representations of images from panel (A), generated using Imaris software.

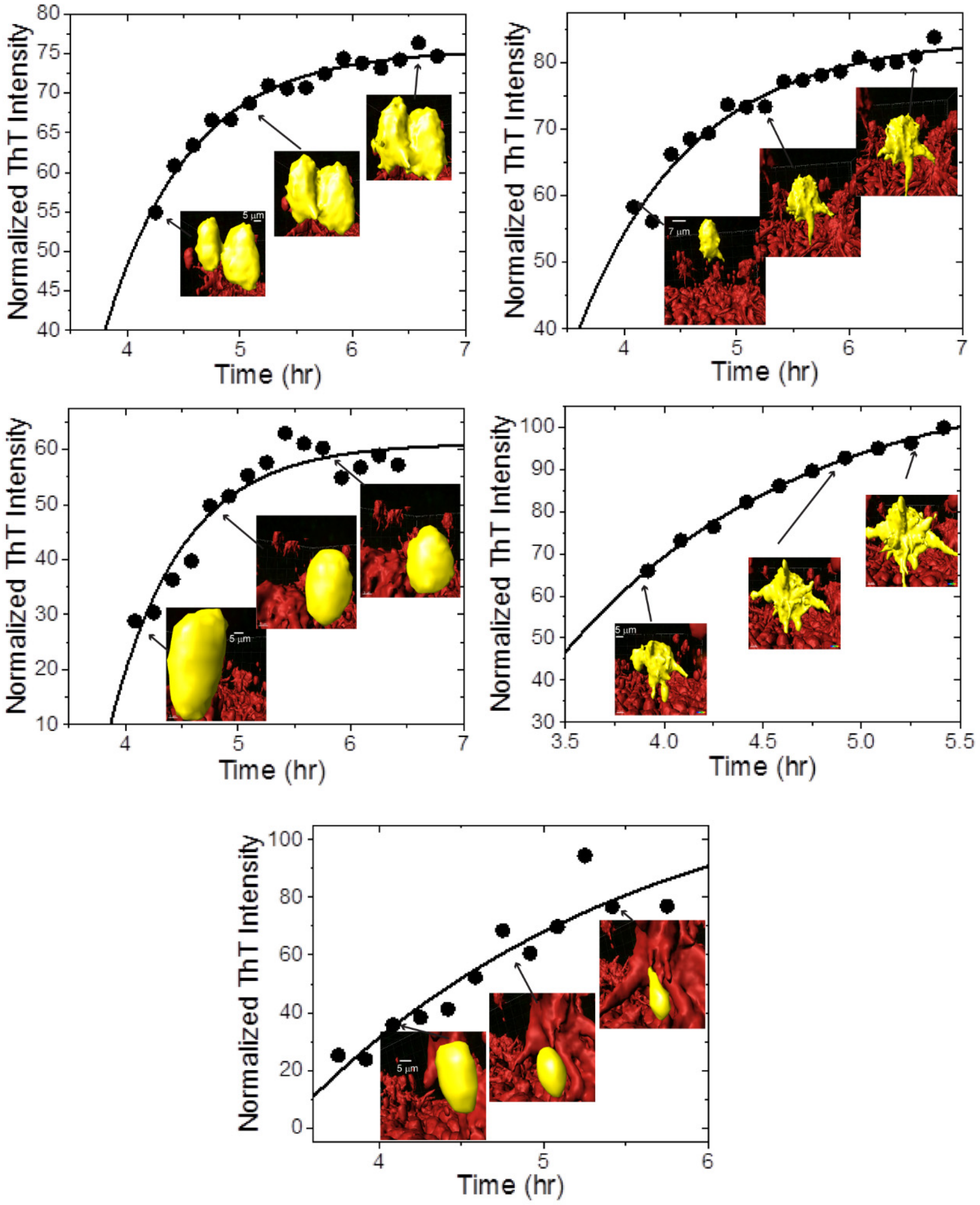

Figure S13. RNA-mediated aging of A1PrD condensates correlates with increased ThT fluorescence. Time-dependent plots of normalized ThT fluorescence intensities (sum of ThT fluorescence intensities divided by condensate surface volumes). Data were fit using a single exponential function (details in Methods).

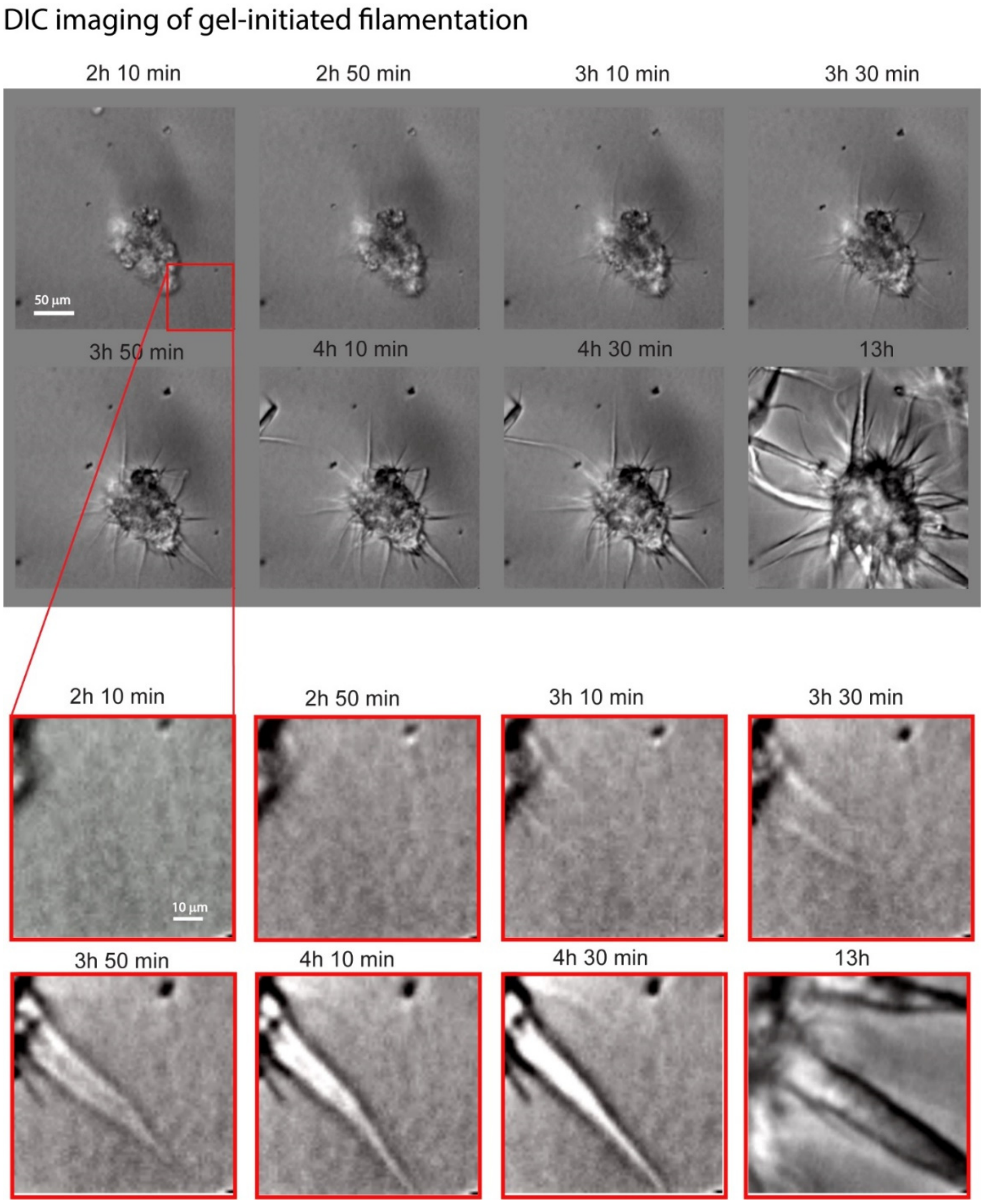

Figure S14. Formation and filamentation of A1PrD starbursts from solid gel clusters. Differential interference contrast (DIC) microscopy images showing different stages of A1PrD gel morphogenesis progressing into starburst aggregates. Conditions: 20 μM unlabeled A1PrD in αβγ buffer (400 mM NaCl, 0 μM RNA).

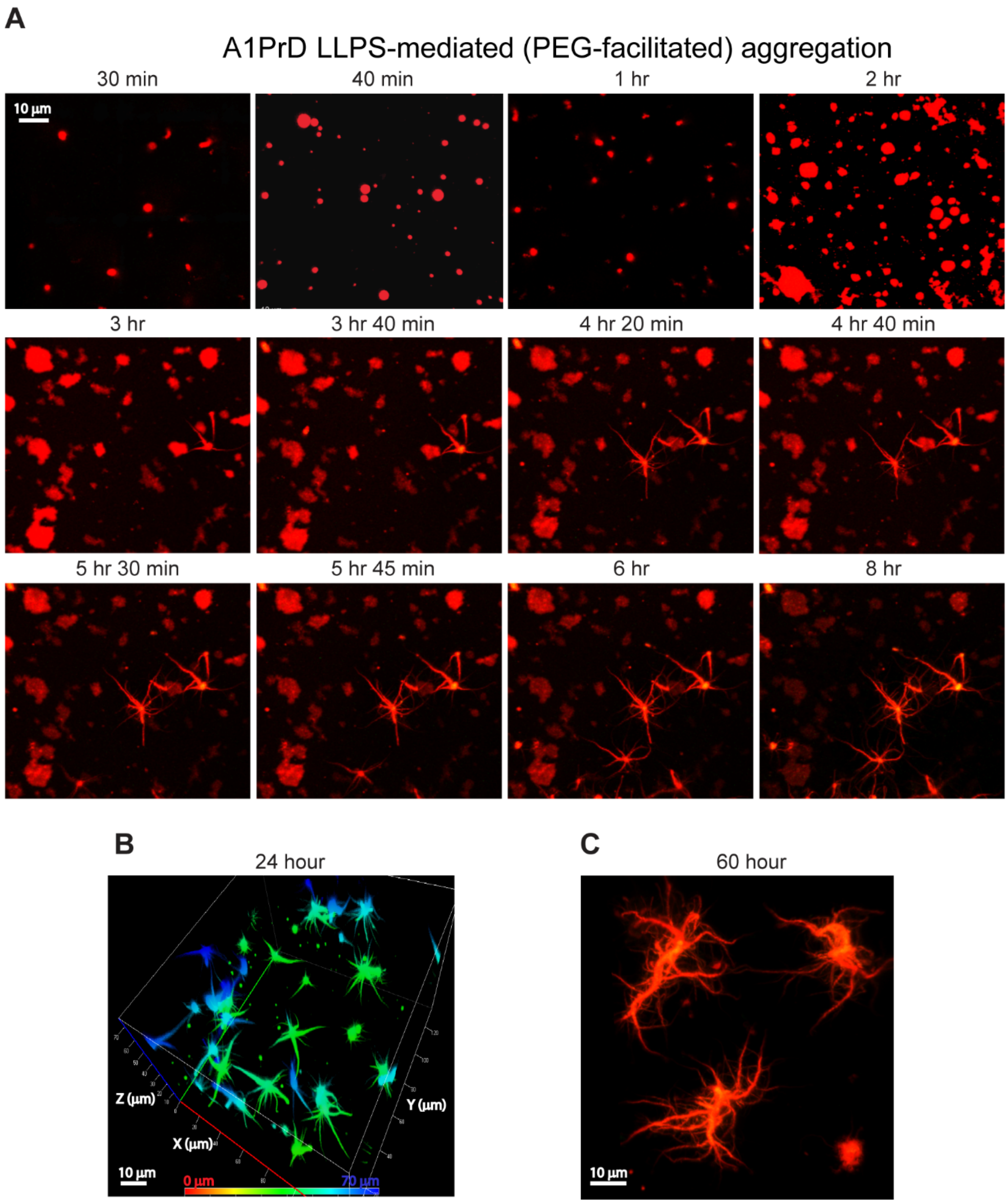

Figure S15. PEG-mediated aging of A1PrD condensates. Crowding-driven protein condensation was examined using 20 μM unlabeled A1PrD, ~70 nM A1PrD-A647, and 10% w/v PEG-8K in αβγ buffer (with 200 mM NaCl unless noted otherwise). (A) Time-lapse confocal microscopy images capturing A1PrD droplet formation, aging, and subsequent starburst development. Droplets formed rapidly (within 30 min), exhibiting spherical morphology with a mean aspect ratio (long axis/short axis) of 1.25 ± 0.2 (n = 65) and diameters averaging 2.4 ± 1.1 μm (n = 65). Larger droplets (>5 μm) frequently demonstrated surface wetting. Images from 20 min to 2 hr represent different regions, whereas subsequent images track the same region. (B) 3D confocal image (after 24 hr) highlighting multiple aging starburst structures. (C) After 60 hr incubation, mature starbursts exhibited extensive networks of secondary ('daughter') spines branching from primary ('parent') spines, resembling neurofibrillary tangle morphologies.

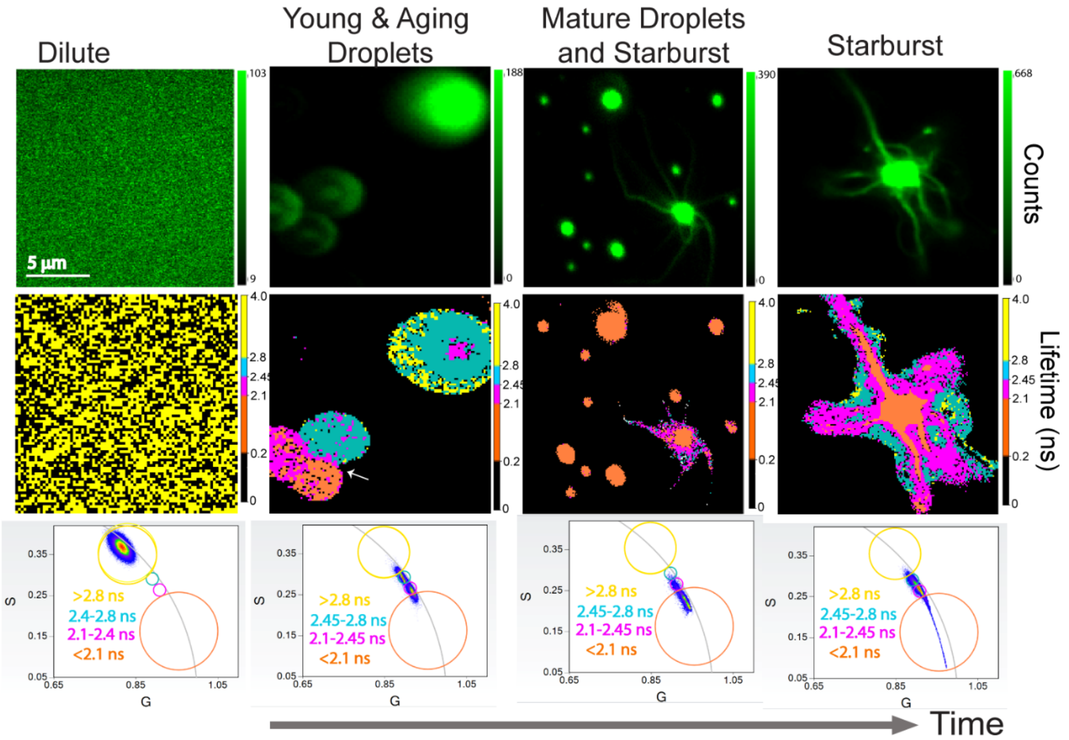

Figure S16. Aging and starburst formation of A1PrD droplets in the presence of PEG. PEG-driven protein condensation monitored using 20 µM unlabeled A1PrD, ~70 nM A1PrD-A488, and 10% w/v PEG-8K in αβγ buffer (with 200 mM NaCl unless otherwise indicated). Representative confocal microscopy (top row) and FLIM images (middle row) illustrate various stages of droplet maturation and starburst formation: (i) non-LLPS dilute conditions; (ii-iv) LLPS conditions at approximately 5 min, 20 hr, and 24 hr post-LLPS initiation. Corresponding phasor plots (bottom row) depict fluorescence lifetimes grouped into four clusters: <2.1 ns (orange), 2.1–2.45 ns (magenta), 2.45–2.8 ns (coral blue), and >2.8 ns (yellow). Numbers within clusters indicate mean phase lifetimes (τψ). Data are based on 48 measurements from two independent replicates.

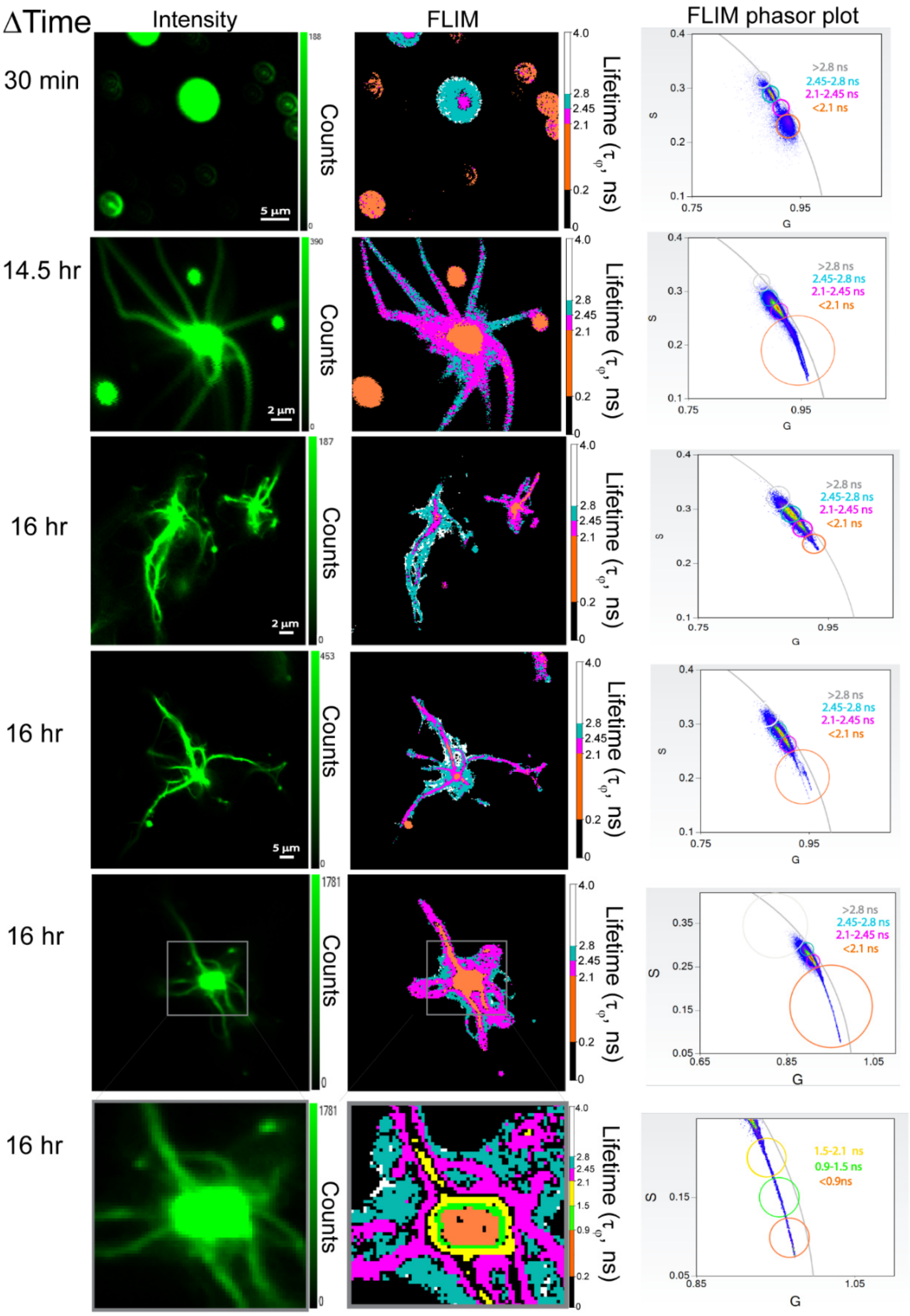

Figure S17. Representative FLIM images tracking PEG-mediated A1PrD droplet aging. FLIM analyses performed on samples containing 20 μM A1PrD, ~70 nM A1PrD-A488, and 10% w/v PEG-8K in αβγ buffer. Shown are confocal microscopy images (left panels), corresponding FLIM images (middle panels), and phasor plots of FLIM data (right panels), illustrating PEG-driven droplet aging and starburst formation. Fluorescence phase lifetimes (τψ) are categorized into four clusters: <2.1 ns (orange), 2.1–2.45 ns (magenta), 2.45–2.8 ns (coral blue), and >2.8 ns (grey). The bottom panels are enlarged views of the boxed regions above (16 hr data), highlighting sub-

clusters with finer lifetime mapping: <0.9 ns (orange), 0.9–1.5 ns (green), and 1.5–2.1 ns (yellow).

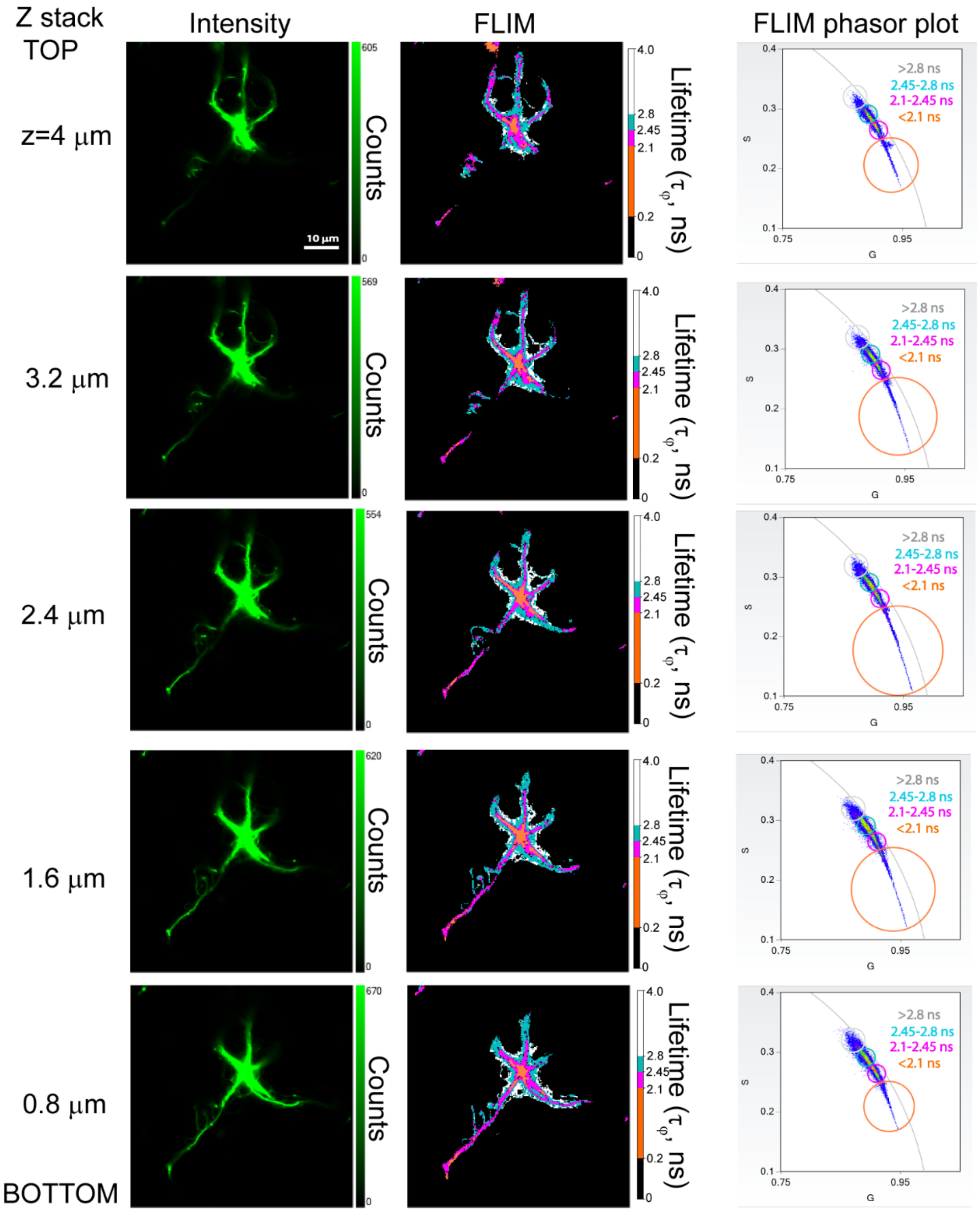

Figure S18. Representative z-stack FLIM images tracking PEG-mediated aging of A1PrD droplets. Confocal microscopy (left panels), corresponding FLIM images (middle panels), and fluorescence lifetime phasor plots (right panels) illustrate PEG-mediated starburst formation from A1PrD droplets. Fluorescence phase lifetimes are categorized into four clusters: <2.1 ns (orange), 2.1–2.45 ns (magenta), 2.45–2.8 ns (coral blue), and >2.8 ns (white/gray).

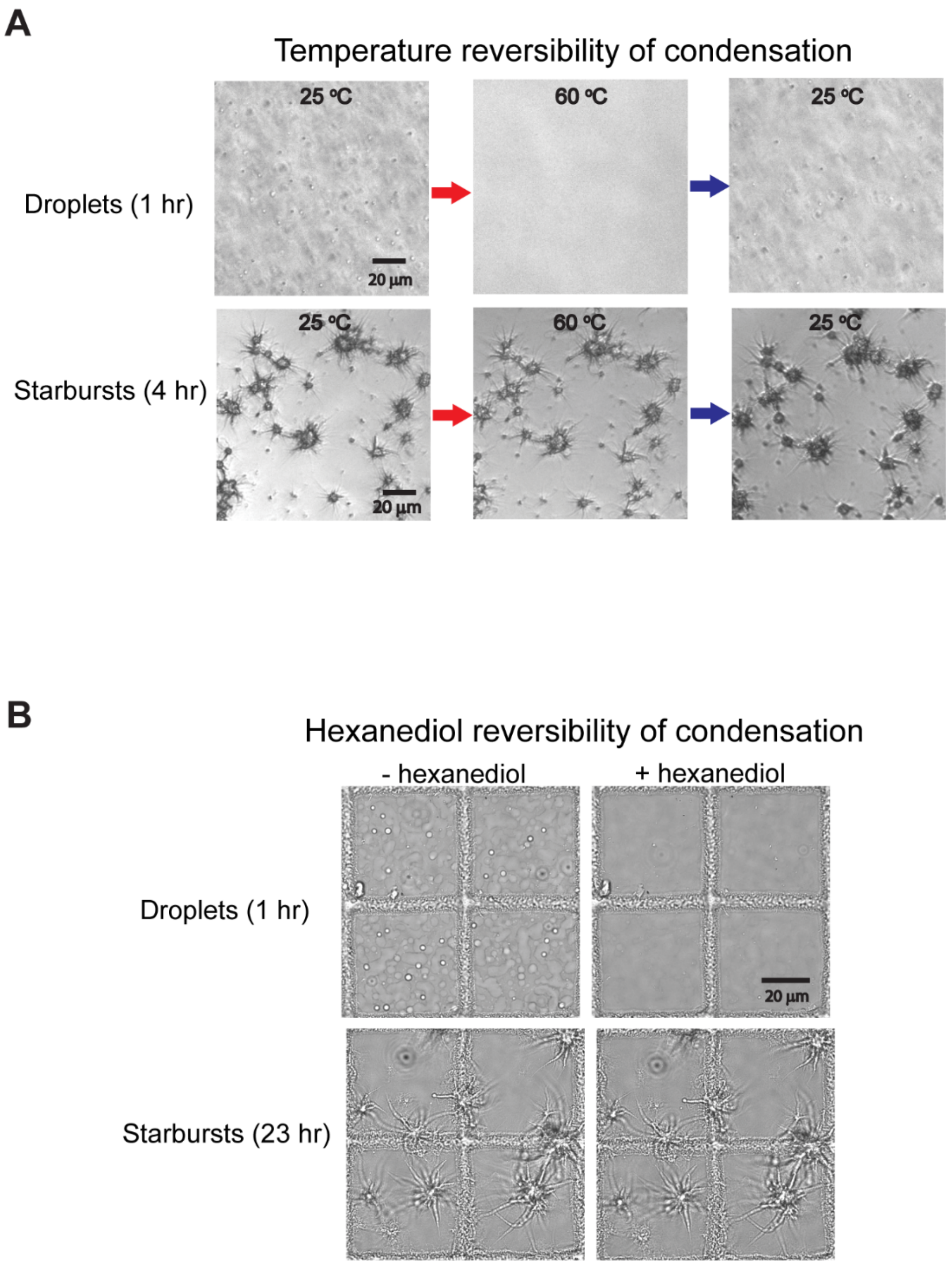

Figure S19. Assessment of droplet and starburst reversibility with temperature and hexanediol. Experiments were conducted using 20 μM A1PrD, 10 μM RNA, and 200 mM NaCl in $\alpha\beta\gamma$ buffer. (A) DIC microscopy images showing heat-induced dissolution of LLPS droplets, whereas starburst structures remain stable. Results are representative of two independent replicates. (B) DIC microscopy images illustrating dissolution of droplets upon addition of 10% 1,6-hexanediol, with starbursts remaining intact. Similar observations were confirmed in two independent replicates.

Movie S1: SDS-mediated dissolution of A1PrD solid gel condensates. Samples containing 20 μM unlabeled A1PrD (with 100 nM A1PrD-A488) under conditions of 0 mM NaCl and 10 μM RNA were incubated for 48 hr. Following addition of 1% SDS (final concentration), confocal microscopy images were captured at 30 s intervals over a period of 3.5 min, demonstrating SDS-induced dissolution of most A1PrD gel condensates.

Movie S2: SDS-mediated dissolution of A1PrD starburst cores. Samples containing 20 μM unlabeled A1PrD (with 100 nM A1PrD-A488) incubated for 48 hr under conditions of 400 mM NaCl and 0 μM RNA. After addition of 1% SDS (final concentration), confocal microscopy images were captured at 30 s intervals over 3.5 min, demonstrating that SDS treatment effectively dissolves starburst cores but leaves fibrillar extensions intact.

Movie S3: Aging dynamics of A1PrD RNA-mediated LLPS and aggregation. Confocal microscopy images capturing an overall view of aged starbursts and residual droplets at 7 hr incubation. Experimental conditions: 20 μM unlabeled A1PrD supplemented with 100 nM A1PrD-A647 and 3 μM ThT, in the presence of 0 mM NaCl and 10 μM RNA.

Movie S4: Starburst initiation, growth through material sequestration, and condensate infectivity as regulated by fluid-solid balance. Time-lapse aerial view (xy plane) capturing the aging of A1PrD droplets and their transformation into filamentous starbursts. The movie begins at 115 min post-incubation, displaying combined green (ThT fluorescence) and red (protein fluorescence) signals. Sample conditions are identical to those described in Movie S2.

Movie S5: Starburst initiation, growth via material sequestration, and condensate infectivity governed by fluid-solid balance. Time-lapse side view (z-dimension) illustrating the aging of A1PrD droplets and their transition into filamentous starbursts. Sample conditions identical to those in Movie S2.

Movie S6: Surface-rendered visualization of starburst initiation, growth by material sequestration, and condensate infectivity. Time-lapse side view (z-dimension) illustrating the aging of A1PrD droplets and their progression into filamentous starbursts. Images are surface-rendered representations derived from Movie S5.

Movie S7: In-phase fusion, siphoning, and infectivity of A1PrD starbursts. Time-lapse side view (z-dimension) showing aging and transition of A1PrD droplets into filamentous starbursts. Surface-rendered images represent a different spatial region than Movies S3–S5, with color coding indicating object mass center positions along the z-axis.

Movie S8: Initiation and growth of A1PrD starbursts without RNA. 3D time-lapse confocal microscopy images (recorded from 70 min to 4 hr at 10 min intervals) depicting the aging of A1PrD droplets and their transformation into filamentous starbursts. Experimental conditions: 30 μM unlabeled A1PrD (with 100 nM A1PrD-A488), 400 mM NaCl, and 0 μM RNA in $\alpha\beta\gamma$ buffer.

Movie S9: Initiation of filamentous starbursts from A1PrD solid gel clusters. Time-lapse DIC microscopy (recorded from 70 min to 13 hr at 10 min intervals), aerial view (xy plane), showing the transformation of A1PrD solid gels into filamentous starbursts. Experimental conditions: 60 μM unlabeled A1PrD, 400 mM NaCl, and 0 μM RNA in $\alpha\beta\gamma$ buffer.

Movie S10: PEG-mediated aging of A1PrD droplets drives filament formation from individual condensates transitioning from liquid to solid phases. Time-

lapse fluorescence confocal microscopy recorded from 70 min to 6 hr at 10 min intervals following the initiation of condensation.

Movie S11: Thermal reversibility of A1PrD liquid droplets. DIC microscopy time-lapse imaging showing dissolution of A1PrD droplets upon heating to 60°C and subsequent reformation upon cooling to 25°C.

Supplementary Figures (Uncropped gel figures)

Figure 2B

[0:0] [0:10] [400:0] [400:10] MW

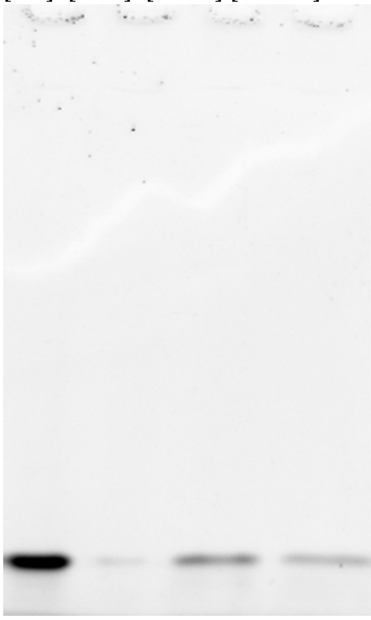

~14 kDa

Figure 2C

[0:0] [0:10] [400:0] [400:10]

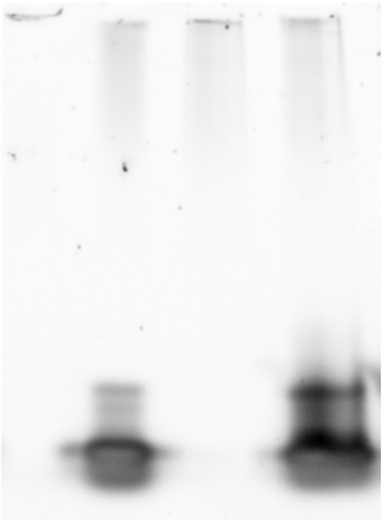

Supplement: Supplementary file 1 [file biomolecules-16-00492-s001.zip › biomolecules-4155527-supplementary/biomolecules-4155527-supplementary.pdf]
